# Intra-strain and inter-strain heterogeneity shape phage-host interactions and phenotypic adaptation in *Pseudomonas aeruginosa*

**DOI:** 10.1128/aem.00050-26

**Published:** 2026-03-03

**Authors:** Bingrui Sui, Xiaoyu Li, Lili Wang, Yongping Xu, Yumin Hou, Muhammad Saleem Iqbal Khan, Na Li, Demeng Tan

**Affiliations:** 1 MOE Key Laboratory of Bio-Intelligent Manufacturing, School of Bioengineering, Dalian University of Technology 592991, Dalian, China; 2 Shanghai Public Health Clinical Center, Fudan University 34748, Shanghai, China; 3 Department of General Surgery, Affiliated Zhongshan Hospital of Dalian University 66562, Dalian, China; 4 Zhongshan Hospital, Fudan University 92323, Shanghai, China; University of Nebraska-Lincoln, Lincoln, Nebraska, USA

**Keywords:** *Pseudomonas aeruginosa*, phage, O-antigen, phage resistance, phage-host interactions

## Abstract

The efficacy of phage therapy relies on a precise understanding of phage-host interactions, particularly how phages recognize bacterial surface receptors and how these interactions shape host adaptation. In *Pseudomonas aeruginosa*, the O-antigen of lipopolysaccharide (LPS) is a common but incompletely characterized determinant of phage susceptibility. Here, we used an O-antigen-specific lytic podophage, phipa9, to prove receptor plasticity in *P. aeruginosa* strain ZS-PA-11. Phage-resistant mutants arose through either a point mutation in *gtaB* (phipa9-G), encoding UTP-glucose-1-phosphate uridylyltransferase, or a large chromosomal deletion (411 kb) (phipa9-B) encompassing *gtaB*. Functional analyses revealed that *gtaB* is essential for O-antigen synthesis, phage adsorption, and infection. Disruption of *gtaB* altered the LPS profile, reduced motility, and promoted biofilm formation. Both resistant mutants (phipa9-G and phipa9-B) exhibited attenuated virulence in *Galleria mellonella*, with the 411 kb deletion mutant showing a more pronounced reduction, underscoring the contribution of O-antigen and deleted loci to bacterial virulence. Comparative assays with another O-antigen-specific phage, phipa10, revealed distinct host ranges and inhibition profiles, highlighting receptor-dependent diversity and possible roles of intracellular defenses. Together, these findings demonstrate that natural variation in O-antigen biosynthetic pathways governs phage susceptibility and bacterial physiology. By uncovering *gtaB*-mediated intra-strain heterogeneity that restricts phage infection while reshaping host phenotypes, this work underscores the dual role of surface receptors in mediating phage-host dynamics and provides mechanistic insight for the rational design of therapeutic phages targeting *P. aeruginosa*.

**IMPORTANCE:**

Effective phage therapy against multidrug-resistant *Pseudomonas aeruginosa* requires understanding how surface receptors govern susceptibility and resistance. We show that *gtaB*-mediated loss of the O-antigen blocks phage adsorption, alters bacterial physiology, and promotes population heterogeneity through pleiotropic phenotypic alterations. These multifaceted consequences reveal that phage resistance is not binary but instead reprograms bacterial adaptation and virulence. Comparison of two O-antigen-dependent phages with distinct host specificities further demonstrates that natural receptor variation critically shapes infection outcomes. Thus, receptor-based resistance represents both a barrier and an opportunity: while it limits phage efficacy, it can also attenuate virulence and expose new vulnerabilities. Recognizing these trade-offs is essential for designing phage therapies that both eradicate pathogens and harness the evolutionary costs of resistance.

## INTRODUCTION

The accelerating spread of multidrug-resistant (MDR) bacterial infections has renewed interest in phage therapy as a precise and evolutionarily adaptable alternative to antibiotics (1). Among gram-negative pathogens, *Pseudomonas aeruginosa* is of particular concern due to its intrinsic resistance, remarkable capacity to acquire additional resistance determinants, and its frequent involvement in chronic and opportunistic infections (2). Although phages infecting *P. aeruginosa* have shown therapeutic potential (3, 4), a critical barrier to clinical translation remains our limited understanding of phage-host interactions. In particular, the identity, diversity, and plasticity of bacterial surface receptors and the mechanisms by which resistance emerges are poorly resolved. Addressing these gaps is essential for designing robust phage therapies and anticipating resistance trajectories in clinical settings.

Phage adsorption to the bacterial surface is the critical first step of infection and is generally mediated by tail fiber proteins that recognize specific host cell envelope structures (5). In *P. aeruginosa*, two of the most common phage receptors are type IV pili (T4P) and lipopolysaccharide (LPS), particularly the highly variable O-antigen component. O-antigen biosynthesis requires coordinated enzymatic activities, including gene *gtaB*, UTP-glucose-1-phosphate uridylyltransferase (6–8). This enzyme generates essential nucleotide sugar precursors for LPS assembly. Mutations in O-antigen biosynthetic genes can provide phage resistance by preventing adsorption. However, such alterations typically incur trade-offs, including reduced bacterial fitness and virulence (7). Notably, structural diversity in O-antigen glycosylation is widespread among *P. aeruginosa* strains. For instance, the laboratory strain PAO1 retains α-1,2-glucose-modified LPS cores, whereas the clinical isolate PA14 accumulates disruptive mutations in LPS core and O-antigen assembly genes. These defects abolish O-antigen production, paradoxically enhance biofilm formation, and shift susceptibility toward phages that target core oligosaccharides (9). Similarly, O-antigen-recognizing phages (e.g., PaoP5 and PaP8) display strain-dependent differences in efficiency of plating (EOP), reflecting the underlying variability in O-antigen structures (10). In addition to point mutations and pathway-specific disruptions, large chromosomal fragment losses, including deletions encompassing *galU*, can also abolish O-antigen production and thereby affect phage infection (11). From an evolutionary standpoint, these contrasting resistance strategies carry different consequences. O-antigen modifications represent a more flexible strategy, but they also come with substantial trade-offs, such as impaired virulence or growth (12). While large-scale chromosomal deletions may provide immediate phage resistance, they often eliminate auxiliary genes and impose severe, pleiotropic physiological defects, limiting their long-term prevalence (13). Importantly, these modifications do not enhance bacterial fitness in the absence of phages, underscoring the evolutionary constraints on resistance strategies. Despite extensive documentation of receptor loss as a mechanism of phage resistance (14), the genetic basis and phenotypic outcomes remain insufficiently characterized in clinical *P. aeruginosa* strains. In particular, how divergence in LPS biosynthetic pathways shapes phage susceptibility and specificity, and whether such receptor mutations could coexist and further drive heterogeneity within bacterial communities, remains poorly understood.

In this study, we isolate and characterize phipa9, a novel N4-like podovirus, and elucidate a defined genetic mechanism of resistance in *P. aeruginosa*. We demonstrate that phipa9 predation drives at least two distinct mutational trajectories: a point mutation in *gtaB* and a large chromosomal deletion encompassing the *gtaB* locus. Both events disrupt O-antigen biosynthesis, effectively abolishing phage plaque formation through structural modification of the LPS. These LPS alterations result in significant pleiotropic phenotypic changes, including impaired motility and attenuated virulence. Unexpectedly, the modified cell surface also promotes enhanced attachment, leading to increased biofilm-forming capacity—a critical trade-off in host persistence. Furthermore, comparative analysis with the O-antigen-dependent phage phipa10 reveals that while both phages exploit the same primary receptor, they possess divergent lytic spectra across clinical isolates. Our findings challenge the convention that phage cocktails should exclusively target non-overlapping receptors; instead, we show that phages binding the same receptor with distinct host ranges can be strategically combined to broaden therapeutic efficacy. Together, these results provide mechanistic insights into O-antigen-mediated resistance and offer fundamental principles for the rational design of next-generation phage therapeutics.

## RESULTS

### phipa9 morphology and genome indicate an N4-like phage

**Fig 1:**
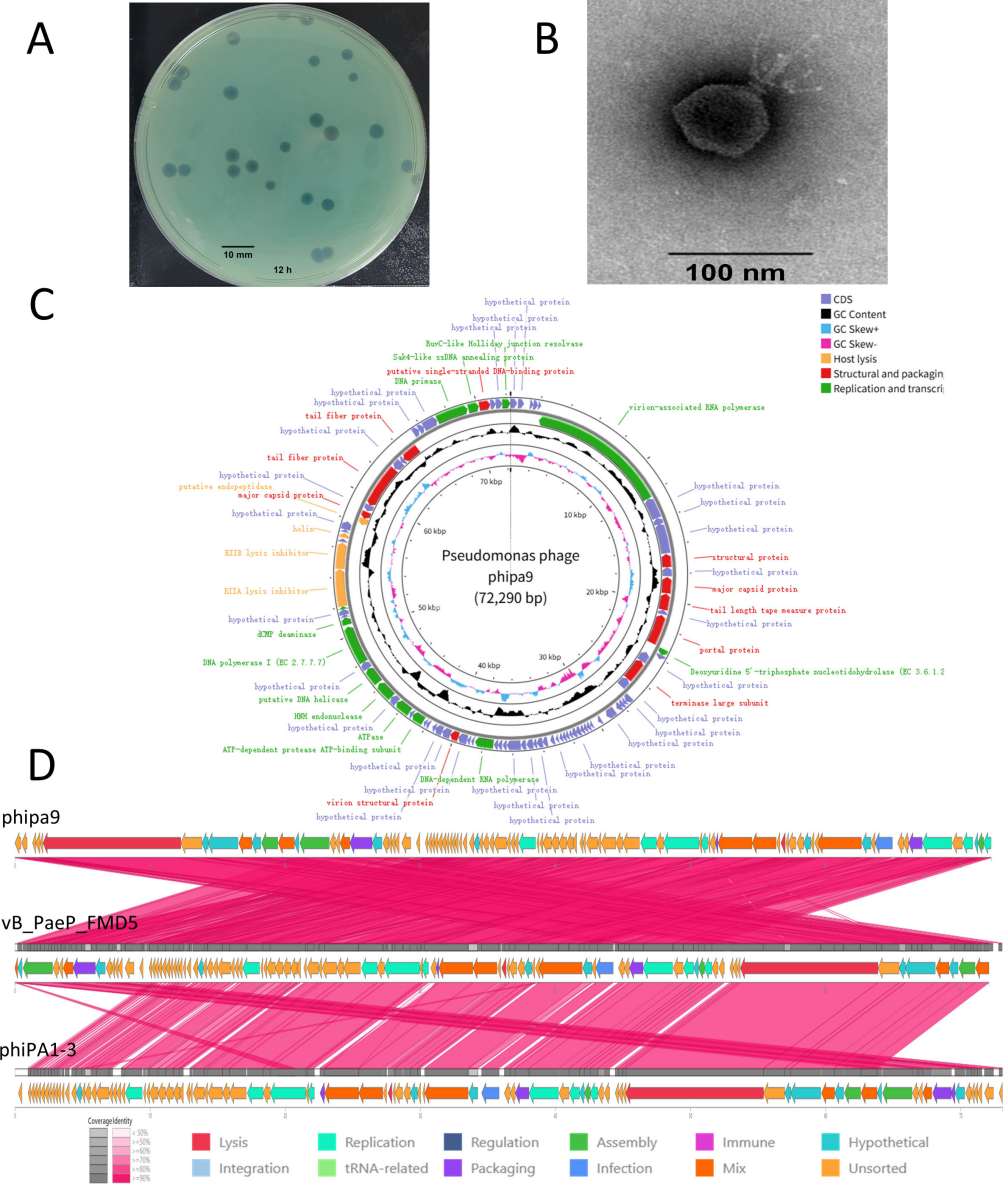
Morphological and genomic features of *P. aeruginosa* phage phipa9. (**A**) Plaque morphology of phipa9 formed on a lawn of *P. aeruginosa* ZS-PA-11 following 12 h of incubation at 37°C. (**B**) Transmission electron micrograph of negatively stained phipa9 virions, showing an icosahedral head approximately 65 nm in diameter. Scale bar, 100 nm. (**C**) Circular genome map of phipa9. The two outermost rings represent ORFs encoded on the forward (outer) and reverse (inner) strands, respectively. The middle black ring denotes GC content, with outward peaks indicating regions above the genomic average. The inner blue and pink rings represent GC skew (G + C/G − C), where blue indicates positive skew and pink indicates negative skew. (**D**) Linear genome alignment of phipa9 with related phages vB_PaeP_FMD5 and phiPA1-3, generated using PhageScope. Arrows represent annotated ORFs, colored according to functional categories. Among them, hypothetical denotes proteins of unknown function and lacking any characterization; unsorted refers to proteins that possess partial annotation but whose modular classification remains ambiguous, precluding confident functional assignment; and mix indicates genomic regions encoding proteins with diverse functional roles. Pink shading connects regions sharing ≥ 75% nucleotide identity.

The lytic phage phipa9, originally isolated from hospital sewage, forms distinct, clear plaques measuring 3–4 mm in diameter on *P. aeruginosa* ZS-PA-11 (Fig. 1A). Transmission electron microscopy (TEM) revealed an icosahedral capsid (~65 nm in diameter) with a short, non-contractile tail, consistent with podovirus morphology (Fig. 1B). Whole-genome sequencing showed that phipa9 possesses a 72,290-bp genome with a G + C content of 55.1%, lower than its host ZS-PA-11 (66.4%). Ninety-one open reading frames (ORFs) were predicted, with ~30% showing homology to characterized proteins. These ORFs are organized into functional modules for replication and transcription, structural and packaging components, and host lysis (Fig. 1C). The replication and transcription module includes thirteen ORFs encoding proteins such as virion-associated RNA polymerase (ORF6), deoxyuridine 5′-triphosphate nucleotidohydrolase (ORF16), DNA-dependent RNA polymerase (ORF48), ATP-dependent protease ATP-binding subunit (ORF58), ATPase (ORF60), HNH endonuclease (ORF62 and ORF69), putative DNA helicase (ORF63), DNA polymerase (ORF65), dCMP deaminase (ORF66), DNA primase (ORF86), Sak4-like ssDNA annealing protein (ORF87), and RuvC-like Holliday junction resolvase (ORF91). The structural and packaging module contains 10 ORFs encoding major capsid protein (ORF12 and ORF77), tail length tape measure protein (ORF13), portal protein (ORF15), terminase large subunit (ORF19), virion structure protein (ORF52), tail fiber protein (ORF79 and ORF82), and putative single-stranded DNA-binding proteins (ORF88). Host lysis is mediated by four ORFs, including RIIA and RIIB lysis inhibitors (ORF70 and ORF71), holin (ORF73), and putative endopeptidase (ORF76). Notably, no virulence factors, antibiotic resistance genes, or tRNAs were detected (Table S1). Based on the phage morphology and ORF similarity, it can be concluded that phipa9 belongs to the N4-like phage family. This classification is supported by its podovirus morphology and the presence of several protein products that are characteristic of N4-like phages, including a large (approximately 3,398 aa) virion-associated RNA polymerase (ORF6), the terminase large subunit (ORF19), and DNA-dependent RNA polymerase (ORF48) (15).

To place phipa9 within an evolutionary context, phylogenetic and comparative genomic analyses were performed. Whole-genome BLASTN alignment indicated that phipa9 exhibits the highest nucleotide sequence similarity to *Pseudomonas* phage phiPA1-3 (GenBank accession OQ378339.1), with 94.5% identity and 99% query coverage. Consistently, genome-based phylogeny positioned phipa9 closest to *Pseudomonas* phage vB_PaeP_FMD5 (97.29% identity with 100% coverage, PP107937.1), indicating strong genomic relatedness (Fig. S1A). All phages in this subclade belong to the taxonomic lineage *Viruses; Duplodnaviria; Heunggongvirae; Uroviricota; Caudoviricetes; Schitoviridae; Migulavirinae;* and *Litunavirus*. Next, phylogenetic analysis based on the large terminase subunit using the maximum-likelihood method further showed that phipa9 terminase clustered with *Pseudomonas* phage phiPA1-3, forming a phiPA1-3–related clade, although the bootstrap support for this node was relatively low (Fig. S1B). In contrast, genome-wide comparisons using VIRIDIC revealed that phipa9 shared an average nucleotide identity of 93.8% with closely related phages and 96.5% identity with *Pseudomonas* phage vB_PaeP_FMD5 (Fig. S1C), exceeding the 95% threshold for species demarcation (16).

Comparative genomic analysis using PhageScope demonstrated high synteny and extensive sequence conservation among phipa9, vB_PaeP_FMD5, and phiPA1-3, particularly in modules related to infection, replication, packaging, and lysis (75%–100% nucleotide identity) (Fig. 1D). Together, these analyses indicate that phipa9 is an N4-like podovirus closely related to vB_PaeP_FMD5, sharing conserved genomic organization and likely similar biological functions.

### A *gtaB* mutation abolishes O-antigen biosynthesis and blocks phage adsorption

**Fig 2:**
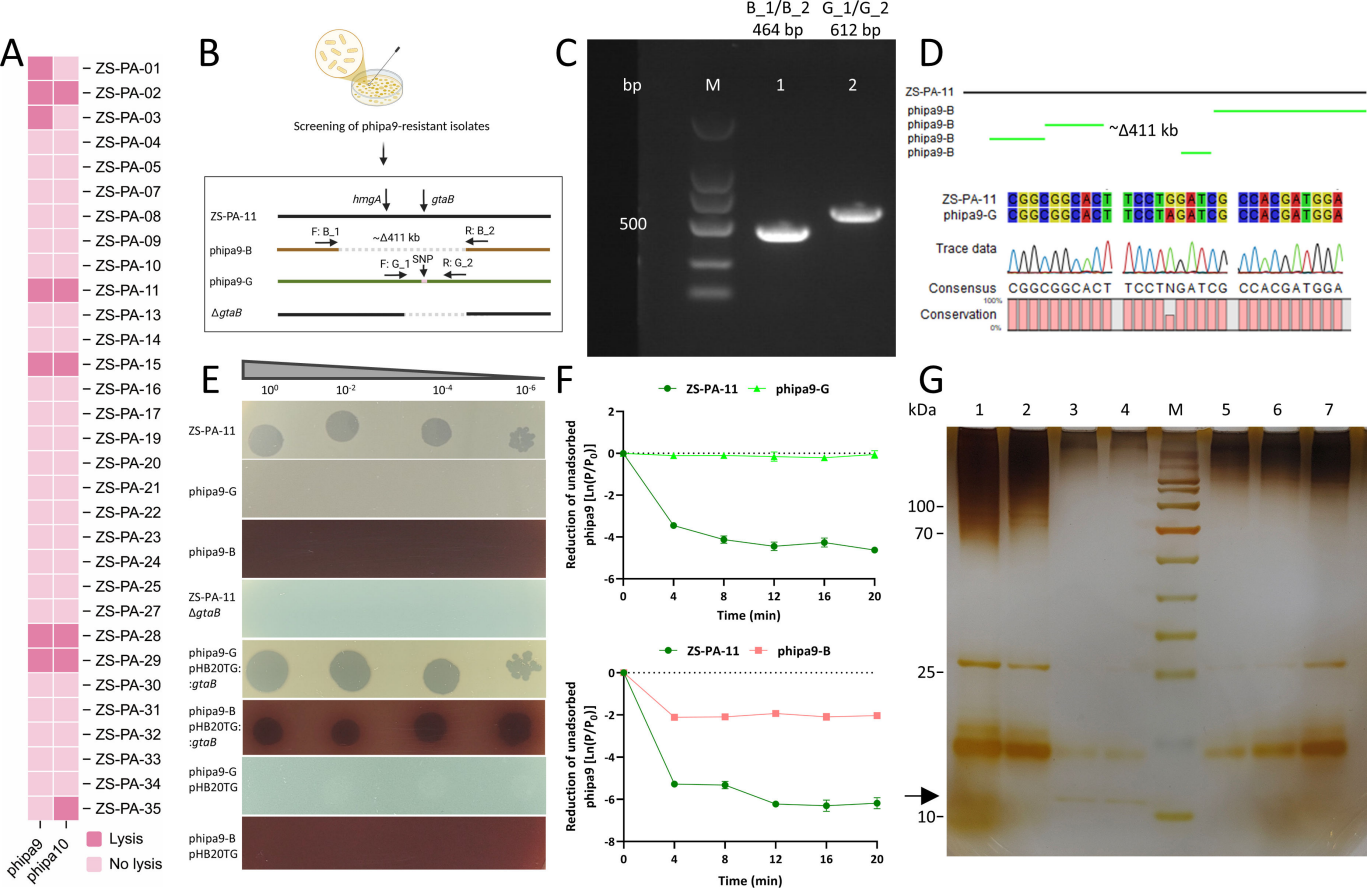
The effect of *gtaB* mutation on phage adsorption and LPS biosynthesis. (**A**) Host range profiling. Infection patterns of phipa9 and phipa10 across the panel of 31 *P. aeruginosa* clinical isolates are displayed as a heatmap. Strains that are susceptible to each phage are marked in dark pink, while resistant strains are shown in light pink. (**B**) Isolation and genetic characterization of two distinct phipa9-resistant mutants derived from host strain ZS-PA-11: (i) phipa9-B, which produces brown pigment and carries a ∼411 kb genomic deletion spanning *hmgA* and *gtaB* (confirmed with primers B_1 and B_2); and (ii) phipa9-G, which harbors a single G→A point mutation in *gtaB*, which was verified using primers G_1 and G_2. (iii) A defined Δ*gtaB* deletion strain was constructed via homologous recombination to further dissect the role of *gtaB*. (**C and D**) Genotype validation by PCR and sequencing. Amplification with primer pairs B_1/B_2 (lane 1 in panel B, ~464 bp) and G_1/G_2 (lane 2 in panel B, ~612 bp) confirmed the presence of the large deletion in phipa9-B and the point mutation in phipa9-G, respectively. Subsequent Sanger sequencing precisely mapped the deletion boundaries in phipa9-B and identified the nucleotide substitution (G→A) in phipa9-G relative to the wild-type sequence. (**E**) Spot assay evaluating phage susceptibility. A 2.5-μL aliquot of a 10⁻²-diluted phipa9 lysate was spotted onto bacterial lawns of the indicated strains. The wild-type strain ZS-PA-11 showed clear lytic zones, whereas the phipa9-G, phipa9-B, and Δ*gtaB* mutants were fully resistant. Phage sensitivity was restored in both mutants upon arabinose-inducible complementation with a wild-type *gtaB* gene (induced with 0.4% L-arabinose). (**F**) Adsorption kinetics of phipa9 to the wild-type ZS-PA-11 and mutants phipa9-B and phipa9-G. The phage adsorbed was quantified over time, demonstrating reduced adsorption to phipa9-B and phipa9-G mutants compared to the wild-type strain. (**G**) Silver-stained SDS-PAGE analysis of LPS extracted from *P. aeruginosa* strains. lanes: 1, ZS-PA-11 (wild-type); 2, Δ*gtaB*; 3, phipa9-B; 4, phipa9-G; 5, phipa10-R; 6, Δ*galU*; 7, and ZS-PA-35 (wild-type). The wild-type strains (lanes 1 and 7) displayed the characteristic smooth-type LPS ladder pattern. Mutants lacking functional *gtaB* (lanes 2–4) or *galU* (lanes 5–6) showed weaker band patterns under the same conditions, including the accumulation of lower-molecular-weight species (arrowhead), indicative of truncated LPS core structures or disrupted O-antigen biosynthesis.

An assessment of the phipa9 host range across clinical isolates revealed a narrow infection profile (Fig. 2A). To contextualize these findings, we employed the previously characterized phage phipa10, which utilizes the O-antigen as its primary receptor, as a comparative reference (11). phipa9 lysed 7 out of 31 tested strains (22.6%; ZS-PA-01, ZS-PA-02, ZS-PA-03, ZS-PA-11, ZS-PA-15, ZS-PA-28, and ZS-PA-29), while phipa10 lysed six strains (19.4%; ZS-PA-02, ZS-PA-11, ZS-PA-15, ZS-PA-28, ZS-PA-29, and ZS-PA-35). Notably, phipa9 failed to infect ZS-PA-35, which was susceptible to phipa10, while phipa10 conversely could not infect two phipa9-sensitive strains, including ZS-PA-01 and ZS-PA-03. This high degree of specificity likely reflects the extensive structural heterogeneity of surface appendages among *P. aeruginosa* strains, which dictates initial receptor recognition. In *P. aeruginosa*, the O-antigen constitutes the outermost glycan layer of the cell envelope, consisting of repetitive heteroglycan subunits anchored to the LPS core. Beyond its role in membrane integrity, this layer functions as a potent physical barrier, shielding underlying outer-membrane proteins from environmental stressors and viral adsorption (17). We first estimated the mutation rate conferring resistance to phipa9, which was approximately 5.91 × 10⁻^6^. Notably, the surviving colonies displayed two distinct colony morphotypes: about 22% exhibited a brown pigmentation, whereas the remainder appeared green on the surrounding agar. From the colonies growing on the plates, 96 isolates were randomly selected for cross-streaking assays. Among them, 47% showed resistance to phipa9, and all brown-pigmented colonies (100%) were completely resistant. Bacterial populations exhibit marked phenotypic heterogeneity following phage infection. While resistant mutants emerge through mechanisms such as point mutations and large deletions, a subpopulation of cells remains susceptible and persists. The mechanism underlying this sustained susceptibility warrants further investigation. Both green and brown resistant isolates were subsequently purified and re-evaluated by spot assays, confirming the acquisition of stable, heritable phage resistance phenotypes. Among the fully resistant mutants (Table S2), two representative phage-resistant variants (designated as “phipa9-B” and “phipa9-G,” respectively) were selected for subsequent experiments (Fig. S2). The phipa9-B mutant exhibits a deletion of ~411 kb, encompassing the *hmgA* gene (homogentisate 1,2-dioxygenase), which regulates the production of the brown pigmentation, and the *gtaB* gene (UTP-glucose-1-phosphate uridylyltransferase), which is required for the biosynthesis of the UDP-glucose precursors necessary for LPS core assembly and O-antigen production (11). While in phipa9-G, we identified a single G→A point mutation at nucleotide 363 in gene *gtaB*, resulting in a glutamine-to-isoleucine (Q→I) substitution in gene *gtaB*, a critical enzyme for O-antigen biosynthesis (Fig. 2B). Both genetic alterations were verified by PCR (Fig. 2C and D).

To confirm that these mutations are associated with phage resistance, an in-frame Δ*gtaB* deletion mutant was constructed in the strain ZS-PA-11. The Δ*gtaB* strain exhibited complete resistance to phipa9, phenocopying the spontaneous phipa9-G mutant and phipa9-B, but its colony morphology remains greenish, unlike that of strain phipa9-B. Complementation of phipa9-G and phipa9-B with a plasmid-borne wild-type *gtaB* restored full susceptibility to phage infection, whereas the empty vector had no effect (Fig. 2E). Previous work has established that *galU* (also known as *gtaB*) is essential for the infection of the closely related *P. aeruginosa* strain ZS-PA-35 by phage phipa10. We therefore hypothesized that mutation of *gtaB* might disrupt LPS structure and hinder phage adsorption. Indeed, phage adsorption assays confirmed that the *gtaB* mutations impaired phage binding capacity, with phipa9 exhibiting significantly higher adsorption to the wild-type strain ZS-PA-11 than to the mutants. The difference was most pronounced at 20 min, with the wild-type adsorbing approximately 80-fold more phage particles than phipa9-G. In contrast, phipa9-B retained partial adsorption capacity, although the wild-type strain still adsorbed 2.5-fold  to  3-fold more phipa9 particles throughout the 20 min (Fig. 2F). Although phipa9-B retained some adsorption efficiency, the complete absence of plaque formation indicates a critical post-adsorption blockade, likely arising from the disruption of LPS-dependent processes required for DNA ejection. Conversely, the point mutation in phipa9-G abolished adsorption by severing initial receptor binding. In addition, silver staining results confirm successful genetic perturbation of LPS biosynthesis in the knockout and mutant strains. Compared with the wild-type strain, the knockout and mutant strains exhibited a reduction or complete absence of specific bands, indicating disruptions in LPS biosynthesis. The concomitant enhancement of the core-lipid A band suggests a blockage in the LPS assembly pathway, preventing the attachment of the O-antigen and leading to the intracellular accumulation of the core-lipid A subunits (Fig. 2G).

Collectively, these findings demonstrate that mutations in *gtaB* drive resistance to phipa9 by compromising LPS structural integrity. Notably, we characterize a bifurcated resistance landscape where the specific genetic architecture of the escape mutation dictates the stage of infection arrest: whereas targeted point mutations in *gtaB* abolish primary receptor recognition and prevent viral adsorption, large-scale chromosomal deletions encompassing the *gtaB* locus yield a distinct phenotype where phipa9 retains high adsorption efficiency yet fails to initiate a productive infection cycle. This divergence suggests that the interaction between phipa9 and its host extends beyond a conventional “lock-and-key” receptor model, revealing a multi-layered molecular basis for bacterial escape that involves both surface-level exclusion and the disruption of downstream, LPS-dependent processes essential for successful viral genome translocation or replication.

### The *gtaB* mutation impairs motility but enhances biofilm formation

**Fig 3:**
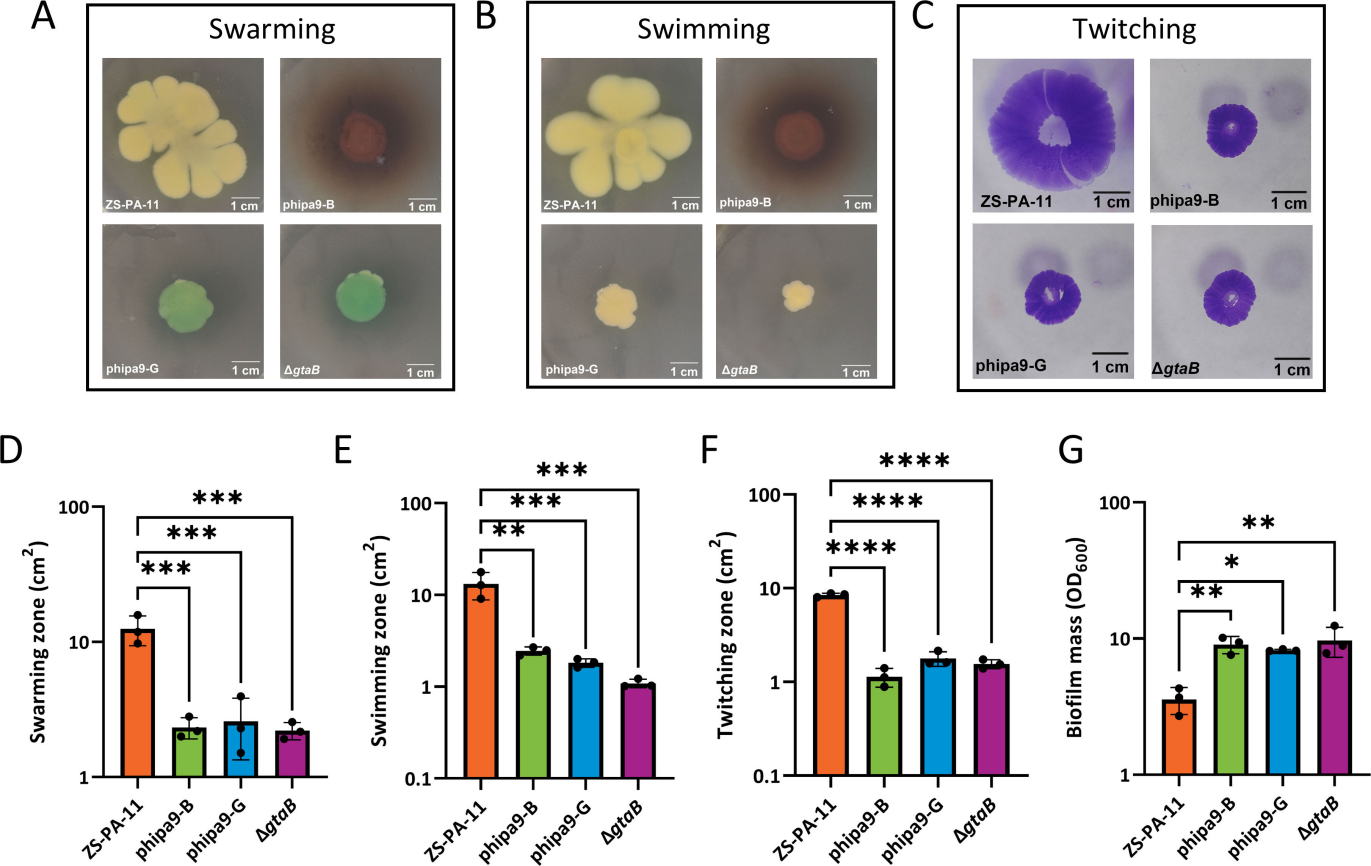
*gtaB*-mediated effects on bacterial motility and biofilm formation. (**A and D**) Swarming motility was assessed by inoculating 2 μL of overnight culture at the center of a 0.5% LB-agar plate and incubating at 37°C for 72 h. The area of the outward-migrating cell front was measured. (**B and E**) Swimming motility was examined by stabbing 2 μL of culture into the center of a 0.5% LB-agar plate and incubating at 37°C for 72 h. The area of the diffuse migration zone was measured. (**C and F**) Twitching motility was evaluated by stabbing through a 1.5% LB-agar layer until contacting the underlying petri dish surface, followed by incubation at 37°C for 72 h. The zone of subsurface migration at the agar-plate interface was measured after careful removal of the agar. (**G**) Biofilm formation was quantified using the tube-adherence assay. Overnight cultures were diluted 1:1,000 in fresh LB and incubated in tubes at 37°C for 7 d without shaking. After washing with water, adherent biofilms were stained with 0.4% crystal violet for 30 min, washed, solubilized with 75% ethanol, and the absorbance at 600 nm was measured. Data are presented as mean ± SD of three biological replicates. Statistical significance was determined by one-way ANOVA. Significance levels are indicated as follows: *P < 0.05, **P < 0.01, ***P < 0.001, ****P < 0.0001.

To assess potential fitness trade-offs associated with phage resistance, we compared motility, biofilm formation, and virulence between wild-type ZS-PA-11 and phipa9-resistant mutants. As expected, both the knockout and mutant strains exhibited a pronounced reduction in motility across all tested movement types. In swarming assays performed on 0.5% LB agar, the knockout strain Δ*gtaB* and the mutants phipa9-B and phipa9-G displayed markedly reduced swarm areas, averaging approximately 1.55, 1.13, and 1.78 cm², respectively, compared to the wild-type strain ZS-PA-11, which achieved a mean swarm area of about 8.46 cm² (Fig. 3A and D, *P* = 0.0003, 0.0004, 0.0004). Swimming motility was likewise significantly compromised in the mutant strains under the same growth conditions. While the wild-type strain achieved an average spread of 12.47 cm², the ∆*gtaB*, phipa9-B, and phipa9-G mutants exhibited drastically reduced migration areas of 2.21 cm², 2.33 cm², and 2.59 cm², respectively (*P* < 0.002 for all; Fig. 3B and E). Similarly, twitching motility on 1.5% agar was severely impaired. The mutants produced mean twitching zones ranging from 1.09 to 2.45 cm², representing a sharp decline compared to the robust 13.2 cm² zone observed in the wild-type strain (*P* < 0.0001; Fig. 3C and F). Taken together, these results demonstrate that *gtaB* is essential for multiple forms of bacterial motility. Importantly, the observed parallel defects in swimming, swarming, and twitching, each mediated by distinct molecular machineries yet collectively contributing to surface-associated translocation, highlight that *gtaB* functions as a central regulator influencing both flagellum-dependent and T4P-driven motility pathways. These results indicate that disruption of *gtaB*, which impairs O-antigen biosynthesis, leads to a pronounced defect in both flagellum- and pilus-mediated motility, highlighting a direct connection between LPS structural integrity and surface-associated bacterial movement.

As O-antigen and LPS integrity are key determinants of biofilm formation and virulence, we next quantified biofilm formation. In contrast to the observed motility defects, the Δ*gtaB*, phipa9-B, and phipa9-G mutants’ strains exhibited significantly enhanced capacity for biofilm formation compared with wild-type ZS-PA-11, producing approximately 2.7-fold, 2.5-fold, and 2.3-fold more biofilm, respectively (Fig. 3G, *P* = 0.0035, 0.0068, and 0.0174). This increase in biofilm biomass may reflect a compensatory adaptation to the reduced motility, as bacteria with impaired dispersal often invest more in sessile growth. The enhancement of biofilm formation in mutants aligns with prior observations linking truncated or altered O-antigen structures to elevated exopolysaccharide production and increased surface attachment in *P. aeruginosa* (11). These findings highlight a clear trade-off associated with phage resistance via *gtaB* mutation: while the loss of functional O-antigen diminishes motility, it simultaneously promotes biofilm formation. Such phenotypic shifts may have important implications for bacterial persistence and pathogenicity, as enhanced biofilm formation can provide protection against environmental stresses, including antimicrobial agents and host immune defenses, potentially offsetting the costs of reduced motility.

### *GtaB*-mediated mutation attenuates *P. aeruginosa* virulence in the *Galleria mellonella* infection model

**Fig 4:**
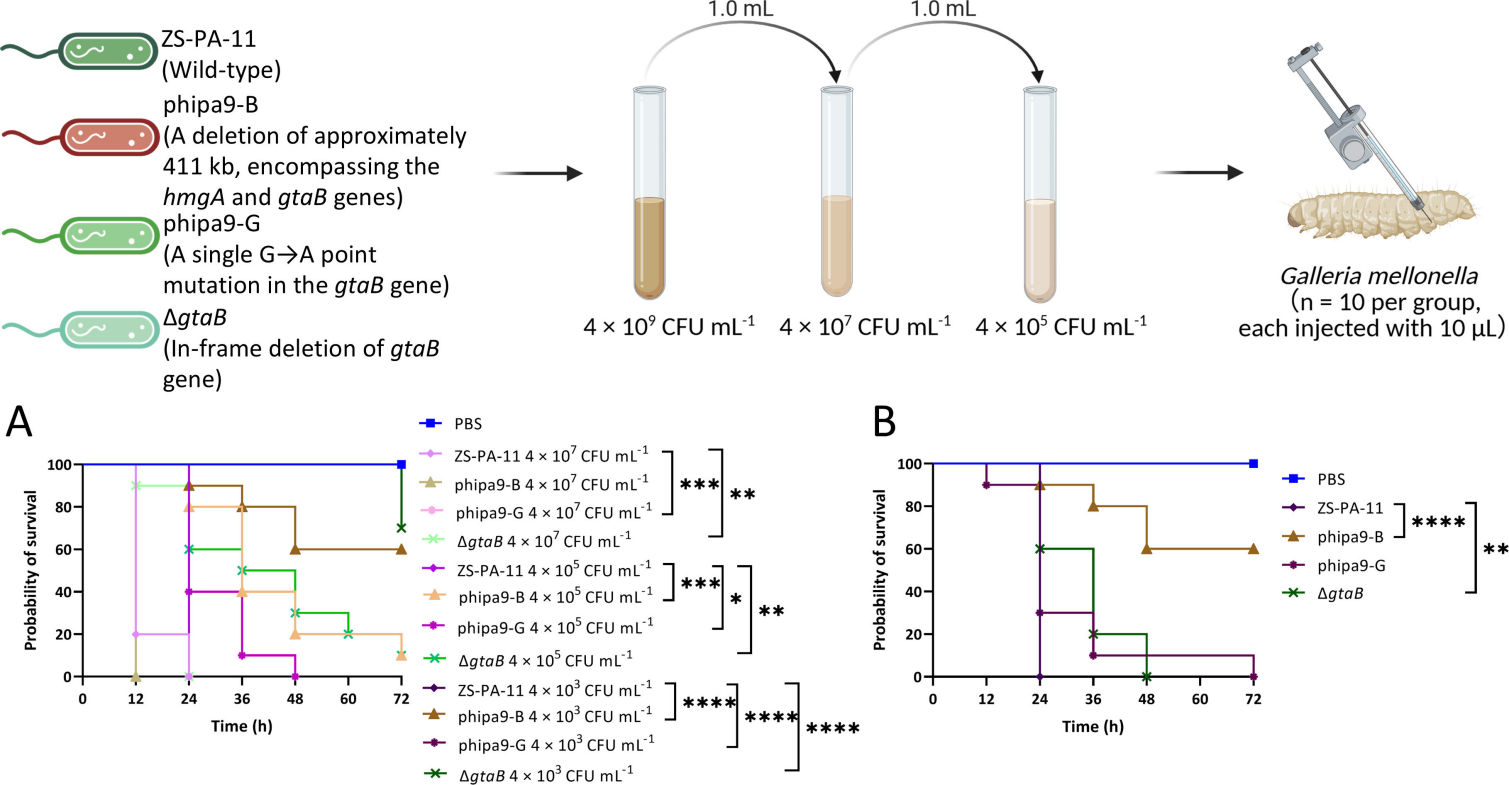
Virulence of *P. aeruginosa* strains in the *G. mellonella* infection model. Larvae (*n* = 10 per group) were injected via the left posterior proleg with 10 μL of bacterial suspension containing 4 × 10^7^, 4 × 10⁵, or 4 × 10^3^  CFU·mL⁻¹ of strain ZS-PA-11 (wild-type), phipa9-B, phipa9-G, or the Δ*gtaB* mutant. Following injection, larvae were maintained at 37 °C in the dark, and survival was recorded every 12 h over a 72-h period. (**A**) Kaplan-Meier survival curves illustrate the survival probability of larvae infected with each strain at three different bacterial doses. Curves were compared using the Log-rank (Mantel-Cox) test and the Gehan-Breslow-Wilcoxon test; statistically significant differences (*P *< 0.05) between each mutant and the wild-type control at the same inoculum are denoted with asterisks in the corresponding panels. (**B**) Virulence comparison at a low bacterial inoculum (4 × 10^3^ CFU·mL⁻¹). The survival profiles of larvae infected with the indicated strains are shown. The Δ*gtaB* and phipa9-B mutants exhibited attenuated lethality compared to the wild-type ZS-PA-11, whereas phipa9-G showed no significant difference. Statistical significance relative to the wild-type control is indicated as follows: *P < 0.05, **P < 0.01, ***P < 0.001, ****P < 0.0001.

The *G. mellonella* larval model is a well-established system for assessing microbial pathogenicity, as its innate immune system recapitulates key features of mammalian innate immunity, including both cellular and humoral defenses (18). Using this *in vivo* model, we assessed whether phage resistance reduces virulence and how distinct genetic alterations, a point mutation in *gtaB* or a large chromosomal deletion, influence *P. aeruginosa* pathogenicity. Larvae were injected with cell pellet suspensions of the wild-type strain ZS-PA-11 or the mutant strains phipa9-B, phipa9-G, and Δ*gtaB* at three inoculum levels (4 × 10^7^, 4 × 10⁵, and 4 × 10^3^ CFU mL⁻¹) and incubated at 37°C for 72 h. Larvae unresponsive to external stimuli were scored as dead. As shown in Fig. 4A, mortality increased with inoculum dose and time post-infection, whereas PBS-injected controls remained fully viable. At the lowest dose (4 × 10³ CFU mL⁻¹), all larvae infected with the wild-type strain succumbed within 24 h, while those infected with mutant strains showed delayed killing; this dose was therefore used for subsequent assays. Under these conditions, phipa9-B, phipa9-G, and Δ*gtaB* caused markedly reduced lethality relative to the wild type (Fig. 4B). At 24 h post-infection, larval survival rates for phipa9-B, phipa9-G, and Δ*gtaB* were 90%, 30%, and 60%, respectively. By 48 h, survival declined to 60%, 10%, and 0%, respectively, still significantly prolonged compared with the wild-type group, in which all larvae were dead by 24 h. Among the tested strains, phipa9-B exhibited the most pronounced attenuation, with 60% of larvae surviving 72 h post-infection, likely due to the extensive genomic deletion in this mutant. Together, these results demonstrate that disruption or loss of *gtaB*-mediated O-antigen variation significantly attenuates *P. aeruginosa* virulence in the *G. mellonella* model. In addition to the O-antigen, genes located within the large chromosomal deletion also contribute to the virulence differences among strains.

### phipa9 exhibits a narrow host range and strain-specific phage productivity in *P. aeruginosa*

**Fig 5:**
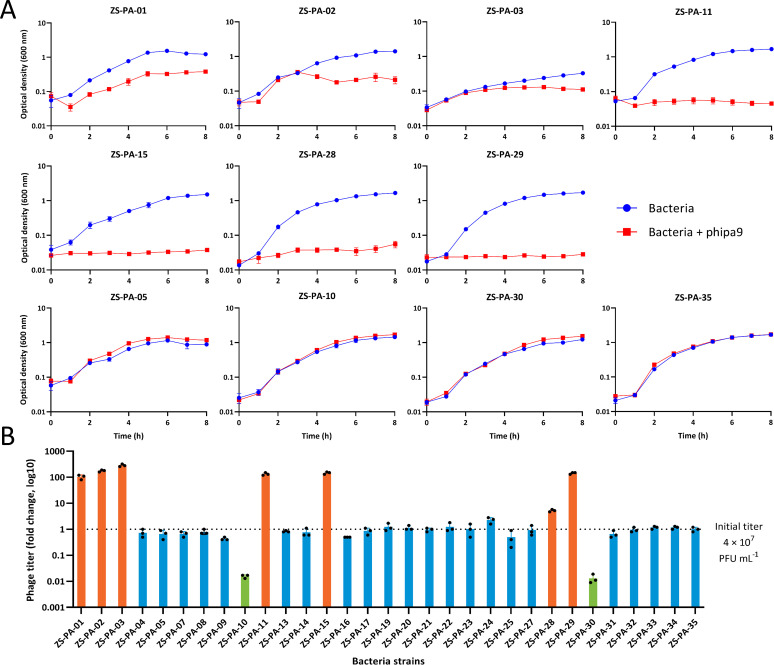
Phage inhibition and progeny production profiles of phipa9. (**A**) Phage-mediated growth inhibition. Bacterial cultures of 11 representative *P. aeruginosa* strains with varying phage susceptibility were co-incubated with phage phipa9 at an MOI of 0.1 in a 96-well plate. Optical density at 600 nm was monitored every 1 h for 8 h using a plate reader. Curves illustrate the differential inhibition of bacterial growth, classified as sensitive (suppression) and resistant (no suppression). (**B**) Progeny phage yield. Following 8 h of co-culture under the same conditions as in panel **A**, samples were centrifuged and treated with chloroform to sterilize residual bacteria. Progeny phage titers were determined by the spot assay on lawns of the indicator strain ZS-PA-11. The bar graph shows the fold change in phage titer relative to the initial inoculum for each of the 31 tested strains. The dashed horizontal line indicates the initial phage inoculum titer (4 × 10^7^ PFU·mL⁻¹). Bars are colored according to three distinct progeny-production categories: orange, phage amplification (fold-change > 1); blue, no net change in titer (fold-change = ~1); and green, suppression of phage propagation (fold-change < 1). Error bars represent the standard deviation of three biological replicates.

Despite the narrow host range of phage phipa9, we sought to investigate its proliferation efficiency across susceptible strains and determine if variations in the EOP influenced phage infection dynamics and production. Following 8 h of co-cultivation, only seven strains (ZS-PA-01, ZS-PA-02, ZS-PA-03, ZS-PA-11, ZS-PA-15, ZS-PA-28, and ZS-PA-29) exhibited significant lytic activity, as evidenced by reductions in optical density (Fig. 5; Fig. S3). To quantify phage production, we measured the release of progeny virions after 8 h of co-culture. Phage production across the seven susceptible strains ranged from a 5.2-fold increase (ZS-PA-28) to a 286.7-fold increase (ZS-PA-03) compared to the starting titer. The original isolation host, ZS-PA-11, exhibited intermediate productivity, indicating that even genetically susceptible hosts can support quantitatively distinct infection outcomes. By contrast, co-culture with non-susceptible strains ZS-PA-10 and ZS-PA-30 produced markedly lower progeny titers, corresponding to only 0.02-fold and 0.01-fold of the initial phage inoculum, respectively. This suggests that perhaps intracellular defense systems may limit phage replication, allowing infection to initiate but aborting the cycle prior to lysis. Indeed, DefenseFinder predicted that both ZS-PA-10 and ZS-PA-30 harbor more than six types of defense systems, including Lamassu-Fam, Prometheus, Septu, BREX, Gao_Qat, gcu24, Rst_3HP, and AbiD (Table S3). Comparative analysis revealed that these systems are also present in other bacterial strains, making it difficult to pinpoint which specific mechanism is responsible for the marked decrease in phage titer. The exact underlying mechanism, therefore, requires further investigation. These differences likely reflect multiple factors, including heterogeneity in O-antigen structure, receptor density and accessibility on the cell surface, and intracellular replication dynamics. Strain-specific variability in phage productivity has important implications for clinical application. Low-yield hosts may act as ecological sinks, reducing overall phage amplification, whereas high-yield hosts can promote robust propagation and effective bacterial clearance. Consequently, reliance on plaque assays alone may overestimate therapeutic potential, highlighting the need for quantitative assessment of phage replication in clinically relevant isolates. Overall, phipa9 exhibits a narrow host range with pronounced strain-specific differences in replication efficiency, emphasizing the critical roles of O-antigen-mediated adsorption and intracellular factors in shaping infection outcomes. This variability underscores the importance of personalized phage selection for effective therapeutic interventions.

### Receptor-specific O-antigen dependencies determine the host specificity of phipa9 and phipa10

**Fig 6:**
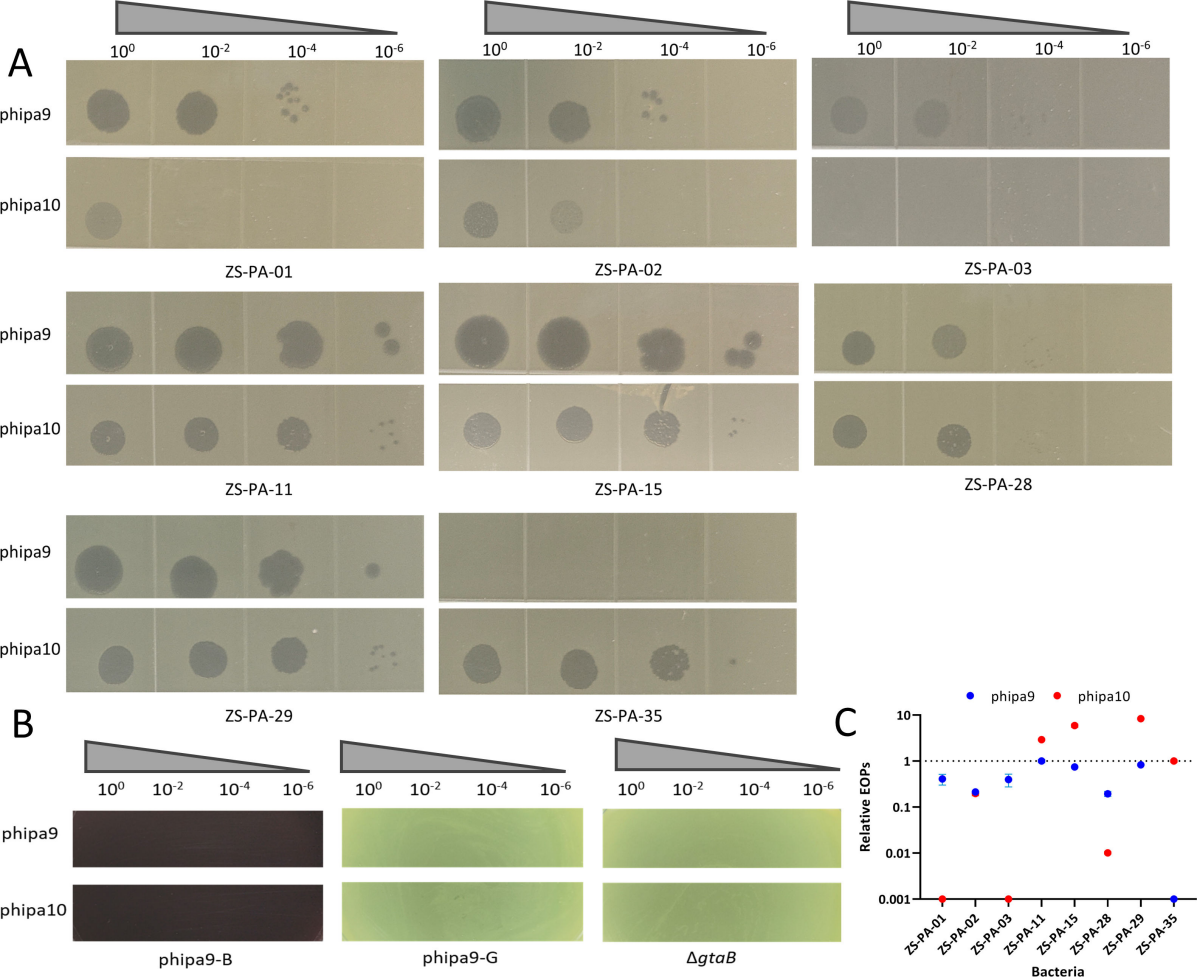
Diversity in receptor binding and host range of phages phipa9 and phipa10. (**A**) Plaque morphology variation. Images of plaques formed by phipa9 (upper row) and phipa10 (lower row) on eight selected sensitive strains after 18 h of incubation at 37°C. Differences in plaque size and clarity reflect strain-specific variations in the phage infection cycle. (**B**) Spot-test assay on receptor-related mutants. The 100-fold serial dilutions (10⁰–10⁻⁶) of phage lysates were spotted onto lawns of the ZS-PA-11 mutants (phipa9-B, phipa9-G, and Δ*gtaB*). The absence of lytic zones reveals that these mutations disrupt the integrity of the gene function, thereby conferring complete resistance. (**C**) Relative EOP. The EOP of each phage was determined on all susceptible strains and normalized to its respective optimal host (phipa9: ZS-PA-11; phipa10: ZS-PA-35). The results represent the mean from three independent assays, with error bars indicating standard deviation.

To investigate the role of O-antigen diversity in phage infectivity, we compared the lytic efficacy of phages phipa9 and phipa10 across eight *P. aeruginosa* clinical isolates as well as derivative mutants of the wild-type strain ZS-PA-11 (phipa9-B, phipa9-G, and Δ*gtaB*). Both phipa9 and phipa10 formed distinct plaque morphologies across the strain panel, and neither produced plaques on the mutant derivatives of ZS-PA-11 (Fig. 6A and B). Given that plaque morphology is a multifactorial phenotype influenced by both biological and technical parameters, these differences may reflect, among other factors, variations in host surface structures or intracellular environments. Furthermore, to quantitatively compare the lytic efficiency of the two phages across different bacterial hosts, we measured their EOP using the double-layer agar method. As shown in Fig. 6C, the EOPs were normalized to the optimal propagation host for each phage: ZS-PA-11 for phipa9 and ZS-PA-35 for phipa10. phipa9 achieved the highest efficiency on ZS-PA-11, with relative EOP values for other susceptible strains ranging between 0.1 and 1. In contrast, phipa10 exhibited even greater efficiency on three strains, namely ZS-PA-11, ZS-PA-15, and ZS-PA-29, than on its reference host, ZS-PA-35. Moreover, adsorption kinetics demonstrated that within 10 min, phipa9 exhibited rapid and efficient adsorption (>90%) to strains ZS-PA-01, ZS-PA-11, ZS-PA-15, ZS-PA-28, and ZS-PA-29, whereas adsorption to the remaining three strains did not exceed 40%. In contrast, phipa10 showed >90% adsorption to ZS-PA-01, ZS-PA-11, and ZS-PA-15, moderate adsorption (>60%) to ZS-PA-28 and ZS-PA-29, and low adsorption (<45%) to the other three strains (Fig. S4). Notably, both phages displayed consistently low adsorption to ZS-PA-03, which may be attributable to the slower growth rate of this strain, potentially limiting the abundance or accessibility of phage receptors during the assay period. Overall, these results indicate that O-antigen heterogeneity among clinical *P. aeruginosa* isolates is a major factor influencing phage susceptibility and contributes significantly to receptor-phage pairing. Pathway-specific dependencies further shape the distinct host ranges of phages phipa9 and phipa10, although post-adsorption host defenses may also modulate infection outcomes.

## DISCUSSION

**Fig 7:**
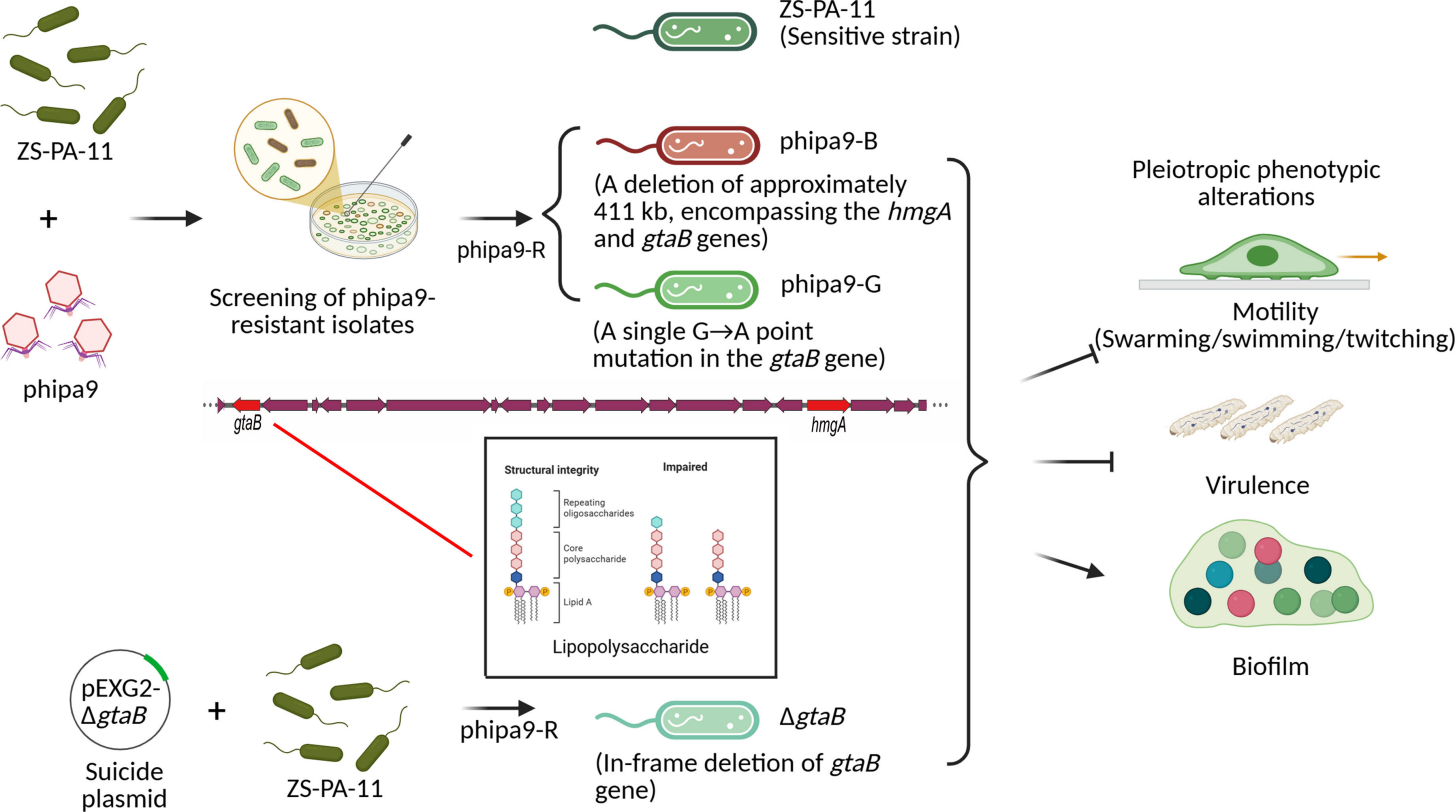
Schematic summary of phage-host interactions revealed in this study. Co-culture of *P. aeruginosa* ZS-PA-11 with phipa9 yielded two distinct resistant mutants: phipa9-B, carrying a ~411 kb deletion including *hmgA* and *gtaB*, and phipa9-G, containing a point mutation in *gtaB*. Both mutants, along with a Δ*gtaB* knockout strain, exhibited impaired motility (swarming, swimming, and twitching), disrupted LPS biosynthesis, and attenuated virulence in *G. mellonella*. In contrast, all *gtaB*-disrupted strains showed enhanced biofilm formation compared to the wild-type strain.

Phages deploy highly specific strategies to recognize and infect their bacterial hosts, often targeting surface-exposed structures such as the O-antigen of the LPS, the primary adsorption receptor in many gram-negative pathogens, including *P. aeruginosa* (17). By altering host metabolism, physiology, and genetics, phages drive microbial heterogeneity through multiple mechanisms, generating diversity even within genetically identical bacterial populations (19). This diversity enables survival in dynamic environments and profoundly shapes community structure and function. The extreme structural variability of the *P. aeruginosa* O-antigen is a well-recognized barrier to phage infectivity and a key driver of resistance evolution (20). In this study, we elucidate the mechanism by which the novel N4-like phage phipa9 recognizes the O-antigen of *P. aeruginosa* ZS-PA-11 and reveal how resistance mutations reshape both phage susceptibility and host physiology, as illustrated in Fig. 7. Our findings demonstrate that such mutations drive intra-population heterogeneity and highlight the pivotal role of phage predation in promoting bacterial evolution within microbial communities, an evolutionary dynamic that also poses significant challenges to the effective application of phage therapy.

Our results demonstrate that successful infection by phipa9 is contingent upon intact O-antigen biosynthesis and that phage adsorption is strictly dependent on the activity of the *gtaB* gene. This gene encodes UTP-glucose-1-phosphate uridylyltransferase, an enzyme essential for generating UDP-glucose, a critical precursor in the O-antigen assembly pathway (8, 11, 21). The requirement of *gtaB* for phipa9 adsorption aligns with its role in maintaining the structural integrity of the LPS; specifically, the O-antigen chain serves as the primary receptor. Silver-staining assays confirmed that the *gtaB* mutation leads to a loss of core LPS chains, providing a clear structural basis for the observed resistance. Intriguingly, while the specific inactivation of *gtaB* abolished binding, the deletion of a larger chromosomal fragment harboring the *gtaB* locus did not impair phage adsorption. This discrepancy suggests a complex regulatory or compensatory mechanism. It is possible that the loss of neighboring genes within the larger fragment removes inhibitory factors or triggers an alternative biosynthetic pathway that bypasses the GtaB requirement, ultimately preserving the phage receptor on the cell surface. This mechanism is in line with previous reports that mutations in LPS biosynthesis genes such as *galU*, *wbpM*, and *wzy* can abolish or markedly disrupt O-antigen expression, thereby conferring phage resistance in *P. aeruginosa* and other gram-negative bacteria. In addition to the SNP detected in *gtaB*, we observed a large chromosomal deletion in phage-selected strain ZS-PA-11 encompassing both *gtaB* and *hmgA*; disruption of *hmgA* is known to drive pyomelanin hyperproduction in both laboratory and clinical isolates. Mutations disrupting O-antigen biosynthesis are a common route to phage resistance in *P. aeruginosa*. Loss of the *galU* gene confers resistance to O-antigen-specific phages such as phipa10 (11). Similarly, disruption of *wbpM* in strain PA103 yields mutants with distinct LPS phenotypes, including a variant (PA103 *wbpM-C*) with a truncated LPS core verified by silver staining (22). Consistently, spontaneous resistance to the O-antigen-targeting phage PaP1 arises from loss-of-function mutations in the *wzy* polymerase gene, underscoring the dependence of such phages on intact O-antigen chains for adsorption (20). Altogether, these studies reveal shared strategies of phage resistance through disruption of O-antigen biosynthesis but also underscore pronounced intra-strain heterogeneity in how *P. aeruginosa* adapts to phage predation.

From an evolutionary perspective, phage predation is a powerful driver of bacterial diversification, as recurrent cycles of phage infection and lysis select for resistant host lineages, typically at the expense of traits such as motility and virulence (23). As anticipated, variations in the O-antigen significantly influence multiple aspects of bacterial physiology, extending beyond phage resistance to affect broader cellular functions. Our findings illustrate a clear trade-off associated with phage resistance: disruption of gene *gtaB* confers resistance but attenuates virulence in the *G. mellonella* infection model while simultaneously promoting biofilm formation. The reduced virulence likely stems from alterations in LPS architecture that modulate host innate immune recognition. In *P. aeruginosa*, LPS serves not only as a major virulence determinant but also as a potent activator of both innate and adaptive immune responses (24). Its natural heterogeneity in lipid A and O-antigen composition, including two outer-core glycoforms of which only one binds O-antigen, suggests that structural modifications, particularly in lipid A or the O-antigen side chain, may significantly change how the innate immune system senses and responds to infection, thereby shaping disease pathogenesis. Our analysis further reveals that the motility-biofilm trade-off, characterized by attenuated motility and enhanced biofilm formation, is broadly distributed among *P. aeruginosa* phage-resistant mutants. This phenotype is not unique to *gtaB* mutations; it also manifests in strains harboring large chromosomal deletions. For example, a phage-resistant variant of *P. aeruginosa* ZS-PA-35, characterized by a ~294 kb chromosomal deletion, exhibited significantly diminished swimming and twitching motility. Conversely, this variant demonstrated an approximate 3-fold increase in surface attachment and biofilm biomass compared to the wild-type strain (11). This phenotypic pattern can be explained by an “adaptive remodeling” mechanism involving one or more of the following processes: modification of surface chemistry following LPS/O-antigen loss, which compromises flagellar and pili function while enhancing adhesion; specific genetic alterations that elevate intracellular c-di-GMP levels, a key secondary messenger that represses motility and promotes biofilm synthesis (25); and perhaps reallocation of metabolic resources from energy-consuming motility systems to matrix production (26). Together, these adaptations account for the trade-off observed in *gtaB* mutants, wherein structural changes to LPS attenuate virulence while enhancing biofilm formation and environmental persistence, an evolutionary strategy that prioritizes long-term survival at the expense of acute pathogenicity. However, this trade-off has clear limits: more severe disruptions to the LPS pathway, such as mutations in essential genes including *wzy* or *wbpH*, cause comprehensive membrane dysfunction and ultimately suppress biofilm development (27). LPS, as a critical component of the bacterial surface, undergoes structural alterations due to its loss or mutation, which, in turn, affects bacterial virulence. It has already been proven through literature that *galU* mutations damage flagellar and attachment-related genes, reducing pathogenicity to host bacteria (28). This phenotypic shift underscores the evolutionary trade-offs that accompany phage resistance: while escape from infection may confer a survival advantage under phage pressure, it simultaneously reshapes bacterial behavior in ways that may influence host-pathogen interactions.

Regarding the enhanced biofilm phenotype, we propose that this pleiotropic shift enables bacteria to withstand intense phage pressure by strengthening attachment to host epithelial surfaces, such as those of the urinary tract or bronchial airways, thereby promoting long-term persistence while evading phage predation. At the same time, robust biofilms provide a fertile ground for horizontal gene transfer, accelerating the recombination of accessory genes that facilitate adaptation to complex microbial niches (29). These findings highlight a double-edged outcome of phage-driven evolution, in which mutations that weaken acute pathogenicity can also promote chronic persistence and genetic innovation. To counter this risk, therapeutic strategies could combine phage cocktails targeting multiple resistance pathways or apply antibiotics when the *gtaB* mutation renders cells more susceptible, thereby enhancing the eradication of these otherwise resilient populations. For instance, strategically designed phage cocktails that target non-redundant bacterial receptors effectively suppress the emergence of resistance, achieving ≥ 96% efficacy against *P. aeruginosa* clinical isolates, including biofilm cultures, and performing equally well in an *in vivo* wound model (30). Additionally, a toddler with extensively drug-resistant *P. aeruginosa* sepsis was successfully treated with an 86-day intravenous phage-antibiotic combination, which showed clear *in vitro* synergy. This synergistic treatment prevented the emergence of clinically significant phage resistance, likely by driving phage-induced virulence trade-offs (31). However, other studies have shown that enhanced adhesion does not necessarily translate into improved *in vivo* colonization. For example, phage-resistant uropathogenic *Escherichia coli* frequently carries mutations in LPS biosynthesis genes, which increase adhesion, invasion, and biofilm formation *in vitro* without enhancing colonization efficiency *in vivo* (32). Among the mutants of phage-resistant extra-intestinal pathogenic *E. coli*, 77% exhibited gene mutations associated with the LPS biosynthetic pathway and demonstrated a significant reduction in virulence in mouse models (33). Collectively, these strategically designed phage cocktails combined with antibiotics show strong synergistic effects, achieving broad-spectrum killing of multidrug-resistant bacteria while preventing the emergence of phage resistance. Clinical and experimental evidence demonstrates that such phage-antibiotic synergy, as seen in the successful treatment of extensively drug-resistant *P. aeruginosa*, can suppress resistance through phage-induced virulence trade-offs.

A particularly striking outcome of this study is the contrasting host ranges of two closely related phages. Although phipa9, a podovirus, and the previously described phipa10, a myovirus, are distinct in virion morphology and genomic composition, sharing no detectable sequence similarity, both phages utilize the O-antigen as their adsorption receptor and exhibit partially overlapping host ranges among 31 clinical *P. aeruginosa* isolates. Despite this shared receptor usage, the two phages show clear differences in EOP and plaque morphology, reflecting distinct infection dynamics and binding kinetics. Notably, phipa10 infects both ZS-PA-35 and ZS-PA-11, whereas phipa9 infects only ZS-PA-11. It is likely that phages phipa9 and phipa10 may bind different epitopes, recognize alternative structural motifs, or detect distinct modification states of the O-antigen, thereby generating overlapping yet non-redundant host ranges among clinical *P. aeruginosa* isolates. Indeed, phage host specificity is largely dictated by tail fiber (or spike) proteins, which mediate the initial, reversible adsorption to susceptible bacterial cells (34). Genome annotation revealed that both phipa9 and phipa10 encode tail fiber proteins; however, bioinformatic analyses showed that these proteins share only limited sequence similarity despite both recognizing LPS as the phage receptor. This observation suggests that the phage tail fibers have evolved to engage distinct structural features of LPS in the two strains. Consistent with this interpretation, mutations in *gtaB* truncate the LPS and abolish O-antigen synthesis, thereby blocking infection by both phages. Given that the O-antigen varies extensively in sugar composition, linkage, and polymerization, we speculate that this structural diversity underlies the distinct host ranges of phages phipa9 and phipa10. Silver staining of LPS from their respective hosts further revealed distinct core banding patterns, confirming that subtle biochemical variations in receptor architecture influence phage binding. These findings align with recent evidence that phage receptor-binding proteins (RBPs), while structurally divergent, can recognize overlapping glycosylation motifs, resulting in partially shared but distinct host specificities and infection phenotypes (35, 36). Overall, these observations suggest that structural diversity in RBPs enables the two phages to engage the same O-antigen receptor through distinct recognition strategies. This work highlights that even within a single receptor class, phage-host interactions can display substantial molecular diversity, an important consideration for the design of phage-based therapeutics.

In conclusion, this study broadens our understanding of O-antigen-mediated phage-host interactions in *P. aeruginosa*, revealing the genetic, structural, and phenotypic consequences of receptor-dependent resistance and offering direct implications for phage therapy. First, classifying phages solely by “receptor type” (e.g., as O-antigen-dependent) risks obscuring key molecular determinants of therapeutic coverage. The design of phage cocktails should instead be guided by high-resolution information on RBP–epitope pairing, quantitative EOP measurements, and detailed knowledge of host LPS architecture. Second, structural variation in LPS, driven by distinct O-antigen types, can impose diverse physiological defects and adaptive costs on bacteria. Finally, these mutation-associated pleiotropic effects complicate the evolutionary landscape of phage-host interactions: for instance, long-term exposure may reduce bacterial virulence while enhancing colonization capacity. Strategically combining phages that elicit different adaptive costs could therefore steer the evolution of antibiotic resistance toward more favorable clinical outcomes, such as diminished virulence and reduced adaptability, thereby strengthening the long-term sustainability of phage therapy.

## MATERIALS AND METHODS

### Bacterial strains, plasmids, antibiotics, and phage

The 31 strains of *P. aeruginosa* detected in this study were initially isolated from Zhongshan Hospital, Shanghai, China. The phage mutant strains constructed and the plasmids used in the experiment are detailed in Table S4. To prepare bacteria cultures, 25% glycerol stock cultures were kept at −80°C, inoculated with fresh Luria-Bertani (LB) medium (10 g L^−1^ NaCl, 10 g L^−1^ tryptophan, and 5 g L^−1^ yeast paste) or on 1.5% LB agar plate (15 g L^−1^ agar added to LB medium), and then cultured aerobically for 12 h at 37°C. The appropriate antibiotic concentrations should be added as follows: 15 μg mL^−1^ gentamicin (Gen 15) for *E. coli*, 75 μg mL^−1^ gentamicin (Gen 75), and 25 μg mL^−1^ triclosan (Tri 25) for *P. aeruginosa*. Phages phipa9 and phipa10 were initially isolated from Shanghai Zhongshan Hospital and, respectively, amplified using the *P. aeruginosa* strain ZS-PA-11 and ZS-PA-35 to obtain high-titer phages (11). The phages were then stored in a buffer containing 10 mM MgSO_4_ and 5 mM CaCl_2_ at 4°C prior to use.

### Transmission electron microscope

The morphology of the purified phages was observed using transmission electron microscopy (TEM). The phage lysate was diluted in a buffer (10 mM MgSO_4_ and 5 mM CaCl_2_) to a titer of approximately 10⁸ PFU mL^−1^ to minimize background interference. The grids were negatively stained with 2% sodium phosphotungstate (wt/vol) for 2 min, rinsed with distilled water, and the excess liquid was absorbed with filter paper. The grid was air-dried before observation under a JEM-2100 TEM (Hitachi, Japan) operating at 80 kV.

### Phage genome DNA extraction and sequencing analysis

The genomic DNA of phipa9 was extracted using the Qiagen DNeasy blood and tissue kit (Qiagen, CA, USA). Briefly, individual phage plaques were excised and suspended in buffer, followed by incubation at room temperature for 4 h. The suspension was then treated with 1% chloroform, centrifuged at 12,000 × *g* for 3 min at 4°C. The 450 μL of the supernatant was treated with DNase I (2 U mL^−1^) and RNase A (20 mg mL⁻¹) at 37°C for 90 min. Subsequently, EDTA (0.02 M) and proteinase K (50 mg mL^−1^) were added to the mixture, which was incubated at 56°C for 90 min. Following these steps, the protocol outlined in the kit manual was followed to extract the phipa9 genomic DNA. Finally, whole-genome sequencing was performed at the Chinese National Human Genome Center (Shanghai, China) utilizing the Illumina platform.

The quality of raw sequence reads was assessed using FastQC v0.11.8 (http://www.bioinformatics.babraham.ac.uk/projects/fastqc), followed by trimming and filtering with Trimmomatic v0.39 (http://www.usadellab.org/cms/?page=trimmomatic) (37). Genome assembly was performedon the Galaxy platform (https://phage.usegalaxy.eu/) using SPAdes (Galaxy Version 4.2.0) (38). Gaps within contigs were closed using GapFiller v1.11 (https://www.baseclear.com/genomics/bioinformatics/basetools/gapfiller) (39). Sequencing errors were corrected with PrInSeS-G v1.0.0 (https://updeplasrv1.epfl.ch/prinses/) (40). Phage genomes were annotated using RAST (https://rast.nmpdr.org/) (41–43). Visualization of phage genomes was done using Proksee (https://proksee.ca/) (44). A comparative analysis of the complete genomes and capsid protein amino acid sequences among closely related phages was performed using the NCBI database (data retrieval date: July 1, 2025), The alignment and phylogenetic reconstructions were conducted with the “build” function of ETE3 (v3.1.3) as implemented in the GenomeNet web service (https://www.genome.jp/tools/ete/) (45), followed by visualization and beautification using the tvBOT (https://www.chiplot.online/) (46). Furthermore, the full genomic sequences of phipa9 and the selected phage were compared using PhageScope (https://phagescope.deepomics.org/) (47). The antimicrobial resistance and virulence genes in the phipa9 genome were analyzed using the Comprehensive Antibiotic Resistance Database (CARD, https://card.mcmaster.ca/) and Virulence Factor Database (VFDB, https://www.mgc.ac.cn/VFs/) databases (48, 49). Potential tRNA genes were predicted using tRNAscan-SE (https://lowelab.ucsc.edu/tRNAscan-SE/index.html) (50). The complete genome sequence of *P. aeruginosa* phipa9 is available in GenBank under the accession number PX572542.1.

### Host range determination

The spot test was used to investigate the host range of phages on 31 clinical *P. aeruginosa* strains. Briefly, 200 μL of each tested overnight-cultured strain was mixed with 4 mL 0.5% LB agar and then overlaid on 1.5% LB agar plates. Subsequently, 2 μL of purified phages was spotted on the surface. After overnight incubation at 37°C, the droplets were examined for the presence of a clear zone around the droplet. The appearance of a clear area means that this bacterium is sensitive to the phages and vice versa.

In addition, the inhibition of 31 strains of *P. aeruginosa* by phipa9 was detected by measuring the OD_600_, and phage titers were calculated by the spotting test. Briefly, at a final concentration of ~4 × 10^7^ PFU mL⁻¹ to each well of a 96-well plate containing 100 μL of bacterial culture (MOI = 0.1), the OD_600_ was measured continuously for 8 h to monitor bacterial growth. Simultaneously, phage samples collected at the 8th h were spotted onto a lawn of *P. aeruginosa* strain ZS-PA-11 to observe the release of progeny phages. The fold change in phage concentration was calculated as the ratio of the progeny phage titer measured after 8 h of co-culture to the initial phage inoculum titer. All experiments were performed in triplicate.

### Screening and identification of phipa9-R strains

The spot test was conducted to screen for phipa9-R strains. The wild-type *P. aeruginosa* ZS-PA-11 was inoculated into LB medium at a 1000-fold dilution and incubated at 37°C, 220 rpm until OD_600_ reached 0.6. A 100-µL aliquot of bacterial culture was mixed with high-titer phipa9 (multiplicity of infection, MOI = 10) and co-incubated at 37°C for 1 h. The mixture was spread evenly on a 1.5% LB agar plate, dried, and incubated overnight at 37°C in the incubator. The growth of bacterial colonies on the plates was observed the following day, and the number of colonies on each plate was counted to calculate the phage mutation rate. The experiment was in triplicate. The resistance phenotypes of bacterial colonies were examined using the cross-streak method, and colonies were further purified and confirmed via the spot test. Eight phipa9-R strains were randomly selected, and bacterial genomes were extracted using the Wizard Genomic DNA Purification Kit instructions (Promega, Beijing, Biotech Co., Ltd) and subsequently sequenced using the Illumina Hiseq platform (Sangon Biotech, Shanghai, Co., Ltd) to delineate the genetic changes. Quality control and filtering of raw data were performed using FastQC V0.11.8 (https://www.bioinformatics.babraham.ac.uk/projects/fastqc/) and Trimmomatic V0.39 (http://www.usadellab.org/cms/?page=trimmomatic) to obtain high-quality data (HQ_Data). Valid data from the samples were compared to the reference genome (wild-type strain ZS-PA-11) using the Burrows-Wheeler Aligner (BWA V0.7.17, https://bio-bwa.sourceforge.net/) and the Genomic Analysis Toolkit (GATK V4.1.1.0, https://gatk.broadinstitute.org/hc/en-us) best practices (51, 52). SNPs were filtered using the MarkDuplicates function in the GATK, and the depth of each gene was evaluated using the BEDTools V2.28.0 (https://bedtools.readthedocs.io/en/latest/) (53). Additionally, the impact of SNPs on genes was predicted using the SnpEff V4.3T (https://pcingola.github.io/SnpEff/) with the predicted impact categorized as high, moderate, low, or modifier (54). Additionally, the defense systems were analyzed using the Defense Finder online platform (https://defensefinder.mdmlab.fr/) (55).

### DNA manipulation and complementation of *gtaB* mutants

The construction of a chromosome in-frame deletion in wild-type strain ZS-PA-11 and complementation in phage-resistant strain phipa9-G were performed as previously described (11). Briefly, DNA fragments flanking *gtaB* were PCR amplified from the wild-type strain ZS-PA-11 using primers introducing *HindIII* and *XbaI* sites and an overlap (~20 bp) of the same sequences to construct a chromosomal frame deletion *gtaB* strain, and then, PCR fusion was performed using upstream and downstream DNA sequences as templates. The PCR products were digested with the two FastDigest Restriction Enzymes mentioned above (Thermo Fisher Scientific, CA, USA) and ligated into the appropriate sites of plasmid pEXG2 with T4 DNA ligase (Thermo Fisher Scientific, CA, USA). The obtained plasmid was transformed into *E. coli* SM10 using the heat shock method and then conjugated into *P. aeruginosa* ZS-PA-11. Transconjugants were initially screened on 1.5% LB agar plates containing Gen 75 and Tri 25. All inserted mutants were cultured on low-nutrient 1.5% agar plates (5 g L^−1^ peptone, 1 g L^−1^ yeast extract, and 15 g L^−1^ agar) containing 10% (wt/vol) sucrose at room temperature for 24 h, and then, the expected mutants were obtained through PCR identification and sequencing verification. Additionally, both mutant strains were verified by PCR amplification, and all primers used are listed in Table S5.

The complete *gtaB* gene of wild-type ZS-PA-11 was amplified by PCR and inserted into the phipa9-B and phipa9-G strains. The complementary plasmid was constructed in the same way as above, except that the FastDigest Restriction Enzymes were replaced by *EcoRI* and *XbaI*, and the plasmid by pHB20TG. The constructed successful plasmid was transferred by conjugating into phage-resistant mutant strain phipa9-G and screened on 1.5% LB plates containing Gen 30 and Tri 100. Transformants that acquired a recombinant plasmid, or the empty vector, were screened for EOP on the double-layer LB plates supplemented with 0.4% L-arabinose (Sigma-Aldrich, St. Louis, MO, USA) to induce the pBAD promoter. In addition, an overnight bacterial culture (ZS-PA-11, phipa9-B, and phipa9-G) was diluted 1:1,000 in fresh LB medium and incubated until reaching an OD_600_ of 0.3. The cells were then serially diluted in PBS, plated (200 µL per plate), and incubated at 37℃ to examine colony morphology.

### Adsorption experiment of phipa9

The adsorption assay of phipa9 to wild-type strain ZS-PA-11 and phage-resistant strain phipa9-G was determined as previously described. In brief, the two strains were cultured overnight in LB medium at 37°C and 220 rpm and then were subcultured at a 1:1,000 dilution into 10 mL of LB until the OD_600_ reached 0.35 (equivalent to 2.6 × 10^8^ CFU mL^−1^). Phage solution (~1.04 × 10^8^ PFU mL^−1^) was added at an MOI of 0.001. Aliquots were taken every 4 min over a 24-min period and centrifuged for 3 min at 4°C and 16,600 × *g*. Unadsorbed phage particles in the supernatant were detected by the double-layer agar method and calculated as described previously (56). Specifically, absorption rates were calculated as the slope of the equation: y = [ln(P_0_) − ln(P)]/B_0_, where P_0_ and B_0_ denote the initial phage titer (PFU·mL^−1^) and bacterial concentration (CFU·mL^−1^), respectively, and P represents the titer of unadsorbed plaques at each time point. Data are presented as the mean ± standard deviation of at least two independent biological replicates.

### Bacterial motility and biofilm formation

To assess the impact of the *gtaB*-mediated mutation on bacterial phenotypes, we evaluated bacterial motility and biofilm formation. For motility assays, overnight-cultured bacteria were adjusted to an OD_600_ of 1.0. For swarming motility, 2 μL of bacterial suspension was inoculated onto the surface of 0.5% soft agar. For swimming motility, the bacterial suspension was inoculated into 0.5% agar plates. After incubation at 37°C for 72 h, motility zones were photographed. For twitching motility, 2 μL of bacterial suspension was inoculated into the center of a 1.5% LB agar plate. After 72 h of incubation at 37°C, the agar was removed and stained statically with 0.4% crystal violet for 30 min at room temperature. After washing off excess stain with tap water, surface displacement was measured to quantify surface motility.

The biofilm experiment was performed according to the methods described in previous literature (11). A 3 μL aliquot of bacterial suspension, adjusted to an OD_600_ of 1.0, was added to 3 mL of LB medium and incubated to allow biofilm formation. After 7 days, the liquid was removed, and 3 mL of 0.4% crystal violet solution was added to each tube for staining. Excess dye was removed by washing three times with tap water. Biofilm quantification was performed by adding 3 mL of 75% ethanol to each tube to dissolve the dye, and the optical density at 600 nm was measured. All experiments were performed in triplicate.

### *G. mellonella* virulence assay

To investigate the impact of the *gtaB* gene deletion on the virulence of *P. aeruginosa*, the experiment was conducted using *G. mellonella*. Ethical approval was not required for the use of *G. mellonella* in bacterial infection experiments. The larvae (weighing approximately 0.1–0.3 g and measuring 2.5–3 cm in length) were placed in sterile plastic Petri dishes, with 10 larvae per dish, and fasted for 24 h prior to inoculation with *P. aeruginosa* to allow acclimatization. Overnight bacterial cultures were transferred to fresh LB medium and incubated to an OD_600_ of 0.5. The bacterial pellet was harvested by centrifugation, washed three times with PBS, and serially diluted in PBS. The final dilution was spread onto 1.5% LB agar plates to determine the precise bacterial concentration. A 10 μL aliquot of the bacterial suspension was injected into the hemocoel of each larva via the tip of the left foreleg using a 300 μL syringe, resulting in final bacterial loads of 4 × 10⁷, 4 × 10⁵, and 4 × 10³ CFU·mL⁻¹ in the larvae. The control group larvae were injected with 10 μL of PBS to assess the potential lethal effects of the PBS solution or the injection procedure. Following injection, the larvae were placed in Petri dishes, maintained on a fasting diet, and incubated at 37°C under standard aerobic conditions. Survival rates were monitored and recorded every 12 h for 72 h. Larvae were deemed deceased if they exhibited dark brown or black coloration, showed no motor response, or failed to react to tactile stimulation. Using Prism software, the survival curves were plotted to show the cumulative probability of survival over 72 h using the Log-rank test or Gehan-Breslow-Wilcoxon test for statistics.

### Detection of O-antigen diversity and EOP analysis

The spot test was employed to investigate the diversity of the receptor O-antigens of phages phipa9 and phipa10. Concisely, 200 μL of the 31 strains and ZS-PA-11 mutant strains (phipa9-B, phipa9-G, and Δ*gtaB*) were combined with 4 mL of 0.5% LB agar and immediately plated onto a 1.5% LB agar plate. Subsequently, a dilution of the phages phipa9 and phipa10 gradient was prepared, and 2 μL was added to the surface. After incubation at 37°C overnight, the plate was observed for the formation of transparent phage plaques. Data visualization was performed using tvBOT (https://www.chiplot.online/).

Adsorption of phipa9 and phipa10 to susceptible strains was assessed as described above, with the following specific conditions: The eight strains were grown overnight in LB medium at 37°C with shaking (220 rpm), diluted 1:1,000 into 5 mL fresh LB, and cultured to an OD_600_ of ~0.35 (~2.6  ×  10⁸ CFU mL⁻¹). Phage was added at an MOI of 0.001. After 10 min of incubation, samples were centrifuged (16,600 × *g*, 3 min, 4°C). Unadsorbed phage in the supernatant was titrated using the double-layer agar assay. The adsorption rate was calculated as: Adsorption rate (%) = [(initial titer – unadsorbed titer)/initial titer] × 100.

Additionally, the EOP of phages phipa9 and phipa10 against 31 bacterial strains was evaluated using a double-layer agar method. Briefly, phage suspensions were serially diluted and mixed with each bacterial strain before being plated on double-layer agar. Following overnight incubation at 37°C, the plaque-forming capacity of each phage on different host strains was quantified. The EOP was determined by calculating the ratio of PFU obtained on each phage-sensitive strain to the PFU obtained on their respective primary host strains.

### LPS extraction and electrophoresis

LPS was extracted from the wild-type strains ZS-PA-11 and ZS-PA-35, knockout mutants Δ*gtaB* and Δ*galU*, and phage-resistant mutants phipa9-B, phipa9-G, and phipa10-R using a commercial kit (EX1740, Beijing Solarbio Science & Technology Co., Ltd) according to the manufacturer’s instructions. Briefly, overnight cultures were diluted 1:1,000 into 20 mL of fresh LB medium and incubated to an OD_600_ of ~ 0.6. Bacterial cells were harvested by centrifugation at 8,000 × *g* for 3 min. The pellet was washed twice by resuspension in 500 μL of Reagent A, followed by resuspension in 500 μL of Reagent B. The suspension was then subjected to ultrasonication (200 W, 10 s on, 10 s off) for 10 min using a sonicator (JY92-IIDN, Ningbo Scientz Biotechnology Co., Ltd.). After centrifugation at 10,000 × *g* for 10 min, the supernatant was collected, mixed with 500 μL of Reagent C by vigorous vortexing for 20 s, and incubated at 66°C for 30 min with occasional mixing. Finally, the sample was kept at 4°C overnight, followed by centrifugation at 3,000 × *g* for 20 min to obtain the purified LPS supernatant. The obtained solution was loaded directly onto a conventional SDS polyacrylamide gel (Sangon Biotech, Shanghai, Co., Ltd) with 12 μL per well of samples in a Protean III device (Bio-Rad, USA) with a 10-toothed comb, and the gels were run in the gel in Bio-Rad Protean III cells at a constant 12 mA in HEPES Running buffer. Silver staining was performed to visualize LPS bands. The gel was removed from the electrophoresis cell, and the gel was washed three times with distilled water (at least 5 min for each wash). Silver staining was performed according to the kit instructions (Sangon Biotech, Shanghai, Co., Ltd), and the gel was imaged underwater.

### Statistical analysis

Statistical analyses were carried out in GraphPad Prism version 9.2 (GraphPad, La Jolla, CA). Values are expressed as mean ± standard deviation (SD). Differences among multiple groups were determined by one-way analysis of variance, and statistical significance was regarded at *P* < 0.05.

## Data Availability

The sequence data of bacteria and phage genomes, ZS-PA-11 (PRJNA1395158), phage phipa9 (PX572542.1), and phipa10 (OK539826), are available in GenBank.

## SUPPLEMENTAL MATERIAL

Supplemental material aem.00050-26-s0001.pdf Figures S1 to S4 and Tables S1 to S5.

ASM does not own the copyrights to Supplemental Material that may be linked to, or accessed through, an article. The authors have granted ASM a non-exclusive, world-wide license to publish the Supplemental Material files. Please contact the corresponding author directly for reuse.
