# Supplementary material for: Intra-strain and inter-strain heterogeneity shape phage-host interactions and phenotypic adaptation in Pseudomonas aeruginosa
Source: Appl Environ Microbiol. 2026 Mar 3;92(4):e00050-26. doi: 10.1128/aem.00050-26 (PMC13101515; doi:10.1128/aem.00050-26)
Supplement: Supplemental material — Figures S1 to S4 and Tables S1 to S5. [file aem.00050-26-s0001.pdf]

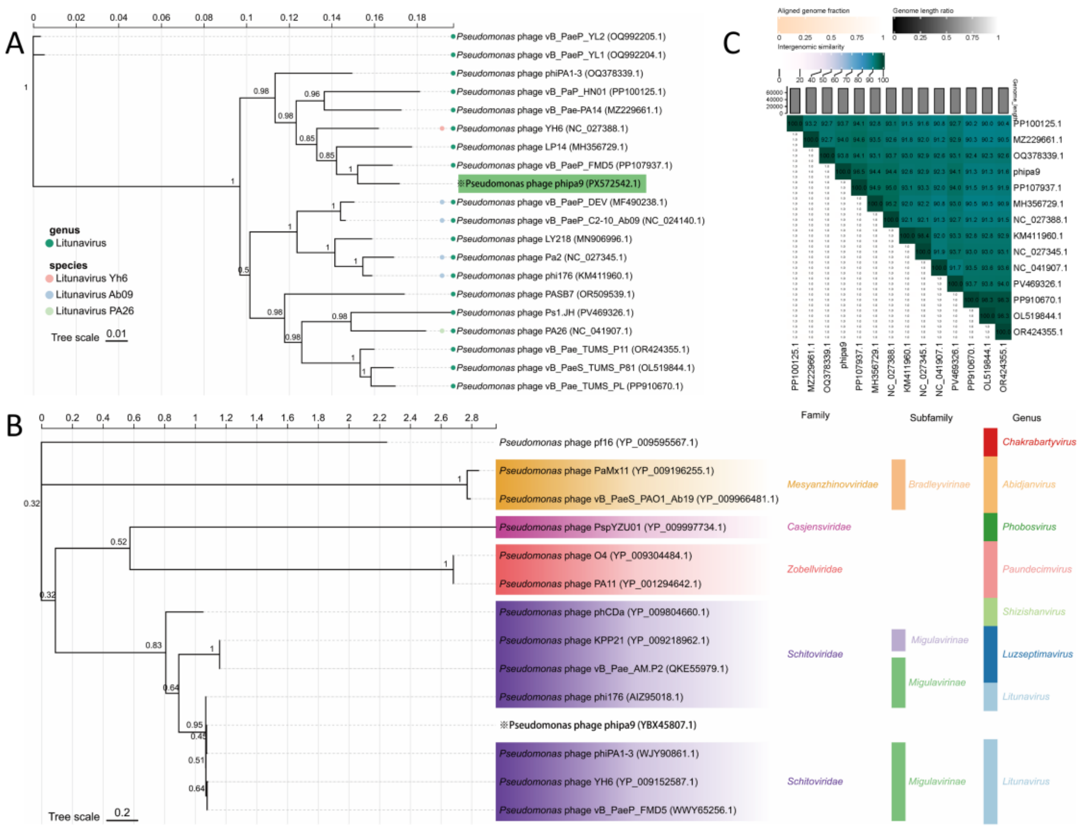

Fig. S1 Phylogenetic and genomic analyses of phage phipa9 and related bacteriophages. (A) Whole-genome nucleotide phylogeny. Relevant sequences were retrieved from the NCBI database. The maximum-likelihood tree was reconstructed in RAxML v8.2.11 using the GTRCAT model with default parameters. Branch support values were derived from 100 bootstrap replicates. (B) Amino acid phylogeny of the large terminase subunit. The tree was inferred in RAxML v8.2.11 under the PROTGAMMALG model, with branch supports assessed from 100 bootstrap replicates. Sequences aligned with MAFFT v6.861b under default parameters, followed by trimming using the gappyout algorithm in trimAl v1.4.rev6. All alignments and preliminary phylogenetic reconstructions were performed via the "build" function of ETE3 v3.1.3 on GenomeNet (https://www.genome.jp/tools-bin/ete). (C) Heatmap of whole-genome Average Nucleotide Identity (ANI) among phipa9 and related phages. Pairwise ANI values were calculated using VIRDIC, and the resulting percentages are displayed in the heatmap to illustrate genomic relatedness.

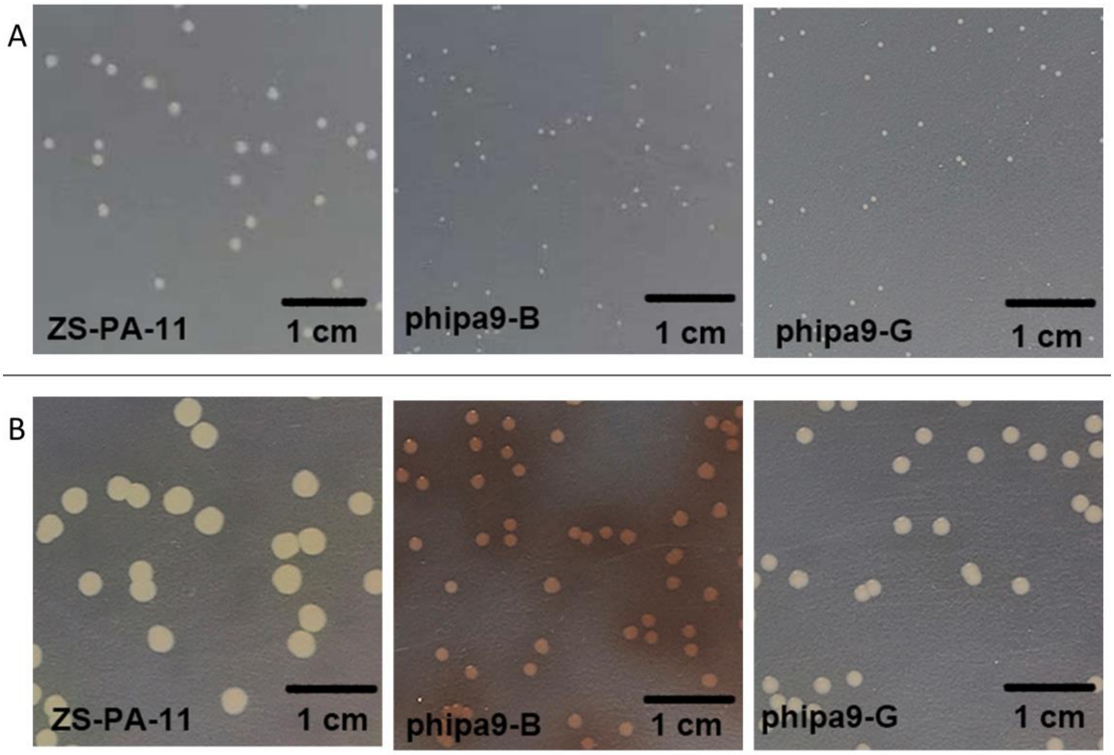

Fig. S2. Comparative colony morphology of *P. aeruginosa* strain ZS-PA-11 (wild-type) and its derivative mutants, phipa9-B and phipa9-G. Cultures were imaged following incubation at 37°C for (A) 18 hours and (B) 42 hours. Both mutant strains consistently produced smaller colonies than the wild-type. Notably, strain phipa9-B exhibited the accumulation of a distinct brown pigment during extended incubation (42 h).

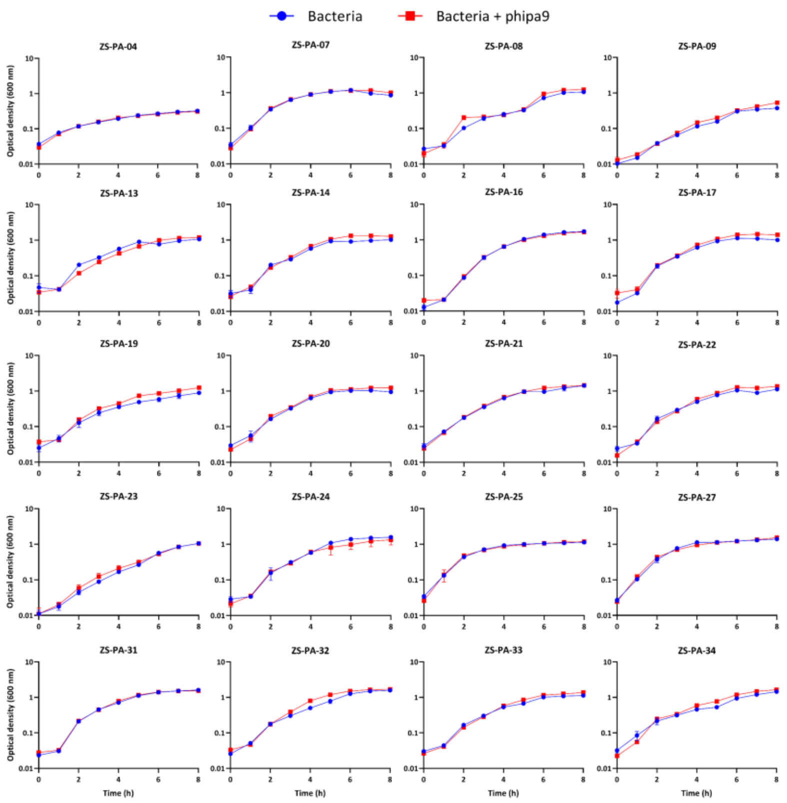

Fig. S3. Inhibition kinetics of phage phipa9 against 20 *P. aeruginosa* strains. The growth curves illustrate the antibacterial efficacy of phipa9 over an 8-hour co-incubation period. Bacterial density was monitored hourly by measuring optical density at 600 nm (OD_{600}).

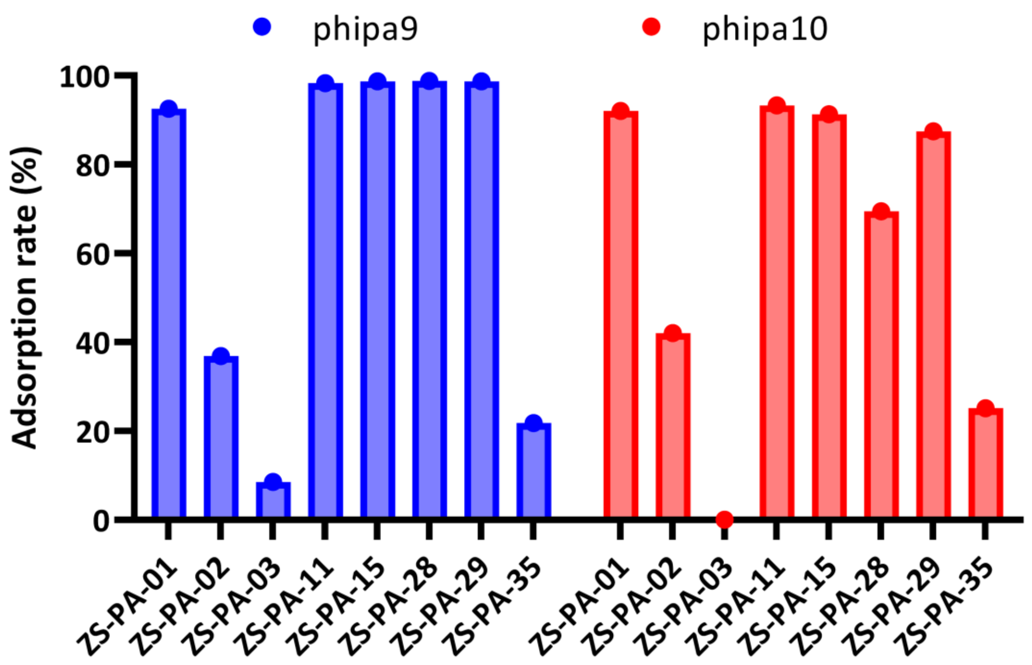

Fig. S4. Comparative adsorption efficiencies of phages phipa9 and phipa10. The bar graph illustrates the adsorption rates of phipa9 (blue) and phipa10 (red) across eight selected *P. aeruginosa* strains, measured 10 minutes post-infection. Data represent the percentage of phage particles successfully bound to the host cells relative to the initial inoculum.

Table S1 Genome annotation of phipa9

ORF	location	Strand	Function	BLAST alignment	Best BLAST Hit Accession	Percent Identity (%)	Query Coverage (%)	Classify
1	1_438	+	hypothetical protein		YP_009152569.1	96.55	100	
2	496_900	+	hypothetical protein		YP_009226136.1	97.76	100	
3	1307_1543	+	hypothetical protein		YP_010659102.1	98.72	100	
4	1540_1857	+	hypothetical protein		XLJ74089.1	98.10	100	
5	1867_2070	+	hypothetical protein		WRN92309.1	97.01	100	
6	12282_2086	-	hypothetical protein	virion-associated RNA polymerase	YP_010658954.1	98.53	100	replication and transcription
7	13848_12283	-	hypothetical protein		XLJ74086.1	99.42	100	
8	14315_13848	-	hypothetical protein		WNV46169.1	98.71	100	
9	16518_14296	-	hypothetical protein	N4 gp53-like protein	YP_009031846.1	99.46	100	
10	17540_16575	-	hypothetical protein	structural protein	WBM84722.1	97.82	100	structural and packaging
11	18209_17544	-	N4 gp55-like protein		UNI71970.1	50.67	99	
12	19465_18266	-	major capsid protein		UVN14359.1	99	100	structural and packaging
13	20690_19500	-	hypothetical protein	tail length tape measure protein	YP_009206217.1	91.50	100	structural and packaging

14	21028_20690	-	hypothetical protein		YP_009031851.1	100	100	
15	23278_21098	-	hypothetical protein	portal protein	WNV46176.1	98.76	100	Structural and Packaging
16	23313_23732	+	Deoxyuridine 5'-triphosphate nucleotidohydrolase (EC 3.6.1.23)		YP_009148261.1	92.81	100	replication and transcription
17	23736_24035	+	hypothetical protein		XSG66949.1	90.91	100	
18	24802_24068	-	hypothetical protein		ANT44452.1	99.49	80	
19	26451_24799	-	terminase large subunit		YP_009152587.1	98.36	100	structural and packaging
20	27179_26451	-	N4 gp69-like protein		WMI40304.1	98.34	100	
21	27246_27677	+	hypothetical protein		WNV49532.1	95.10	100	
22	27677_27889	+	hypothetical protein		WBM84710.1	97.14	100	
23	27886_28161	+	hypothetical protein		WMX17967.1	97.80	100	
24	28137_28445	+	hypothetical protein		WIL00487.1	99.02	100	
25	28648_29310	+	hypothetical protein		WVH07423.1	93.64	100	
26	29727_29981	+	hypothetical protein		WMX17879.1	100	100	
27	30388_30579	+	hypothetical protein		WVH07511.1	95.24	100	
28	30598_30840	+	hypothetical protein		AWY02795.1	95	100	
29	30837_31067	+	hypothetical protein		YP_010659060.1	90	100	
30	31067_31315	+	hypothetical protein		WMX17884.1	100	100	
31	31319_31633	+	hypothetical protein		QWT71760.1	96.12	99	

32	31617_31919	+	hypothetical protein		WMX17886.1	91	100	
33	31923_32126	+	hypothetical protein		YP_010659056.1	97.01	100	
34	32156_32374	+	hypothetical protein		WQA18458.1	97.22	100	
35	32371_32604	+	hypothetical protein		YP_010659130.1	93.51	100	
36	32601_32822	+	hypothetical protein		WJY90789.1	98.63	100	
37	32894_33079	+	hypothetical protein		YP_009152514.1	100	100	
38	33173_33463	+	hypothetical protein		WQA18454.1	98.96	100	
39	33648_33983	+	hypothetical protein		AWY02784.1	98.20	100	
40	33983_34384	+	N4 gp14-like protein		WLJ71012.1	99.25	100	
41	34384_34635	+	hypothetical protein		WLJ70997.1	95.18	100	
42	34635_35075	+	hypothetical protein		QWY17765.1	50	100	
43	35115_35462	+	hypothetical protein		AWY02780.1	89.57	100	
44	35474_36406	+	N4 gp15-like protein		YP_010659045.1	99.03	100	
45	36419_36679	+	hypothetical protein		YP_010658902.1	97.67	100	
46	36714_36998	+	hypothetical protein		YP_003358418.1	97.87	100	
47	37002_37274	+	hypothetical protein		WMX17901.1	96.67	100	
48	37309_38550	+	N4 gp16-like protein	DNA-dependent RNA polymerase	WRN92352.1	97.58	100	replication and transcription
49	38648_38800	+	hypothetical protein		YP_009148204.1	98	100	
50	38873_39097	+	hypothetical protein		XSG67010.1	90.54	100	
51	39101_39727	+	hypothetical protein		YP_009286247.1	96.63	100	
52	39724_4027	+	hypothetical	Virion	YP_01065	94.02	100	structur

	8		protein	structural protein	9172.1			al and packaging
53	40278_40859	+	hypothetical protein		YP_009031802.1	91.19	100	
54	40859_41374	+	hypothetical protein		YP_009031803.1	95.91	100	
55	41371_41595	+	hypothetical protein		YP_010659069.1	97.30	100	
56	41831_42061	+	hypothetical protein		YP_010659176.1	98.68	100	
57	42070_42255	+	hypothetical protein		YP_003358427.1	96.72	100	
58	42314_43147	+	hypothetical protein	ATP-dependent protease ATP-binding subunit	YP_010659072.1	98.92	100	replication and transcription
59	43144_43386	+	hypothetical protein		YP_003358429.1	98.75	100	
60	43418_44488	+	hypothetical protein	ATPase	YP_009598387.1	98.60	100	replication and transcription
61	44499_45017	+	hypothetical protein		ANT44404.1	98.84	100	
62	45017_46243	+	N4 gp25-like protein	HNH endonuclease	YP_009290571.1	94.12	100	replication and transcription
63	46336_47502	+	hypothetical protein	putative DNA helicase	YP_009148216.2	98.20	100	replication and transcription
64	47502_48029	+	hypothetical protein		YP_010659078.1	97.14	100	
65	48029_50644	+	DNA polymerase I (EC 2.7.7.7)		YP_009031812.1	98.16	100	replication and transcription
66	50777_51295	+	Putative deoxycytidyl	dCMP deamina	YP_009226164.1	91.91	79	replication and

			ate deaminase	se				transcription
67	51327_51467	+	hypothetical protein		AXL96679.1	97.83	100	
68	51470_51868	+	hypothetical protein		QWT71798.1	93.94	100	
69	51865_52056	+	N4 gp22-like protein	HNH endonuclease	YP_009152546.1	100	100	replication and transcription
70	52060_54576	+	hypothetical protein	RIIA lysis inhibitor	YP_009286265.1	94.63	100	host lysis
71	54588_56366	+	hypothetical protein	RIIB lysis inhibitor	YP_009031816.1	92.74	100	host lysis
72	56414_56638	+	hypothetical protein		AWY02750.1	97.30	100	
73	56722_57039	+	hypothetical protein	holin	YP_009152550.1	99.05	100	host lysis
74	57089_57280	+	hypothetical protein		XJP46882.1	100	98	
75	57273_57830	+	hypothetical protein		XJP46881.1	91.89	100	
76	58405_57893	-	hypothetical protein	putative endopeptidase	QKE56056.1	67.26	99	host lysis
77	58926_58402	-	Phage major capsid protein of Caudovirales	major capsid protein	YP_010659013.1	97.70	100	structural and packaging
78	59472_58993	-	Putative dUTPase	hypothetical protein	YP_009031824.1	94.97	100	
79	62681_59472	-	hypothetical protein	tail fiber protein	XYH42921.1	94.95	100	structural and packaging
80	63391_62720	-	hypothetical protein		WBM84652.1	90.13	100	
81	63687_63388	-	hypothetical protein		QHZ59490.1	98.99	100	
82	64973_63684	-	hypothetical protein	tail fiber protein	YP_009598408.1	99.53	100	structural and packaging

83	65366_65794	+	hypothetical protein		WRN92310.1	97.89	100	
84	65791_66171	+	hypothetical protein		WWY65191.1	97.62	100	
85	66173_67183	+	N4 gp42-like protein		WMI40395.1	95.54	100	
86	67198_69366	+	hypothetical protein	DNA primase	QKE55957.1	66.34	100	replication and transcription
87	69415_70149	+	N4 gp44-like protein	Sak4-like ssDNA annealing protein	YP_009031833.1	100	100	replication and transcription
88	70176_70922	+	hypothetical protein	putative single-stranded DNA-binding protein	YP_009031834.1	97.98	100	structural and packaging
89	70926_71291	+	hypothetical protein		YP_009219056.1	98.35	100	
90	71288_71731	+	N4 gp48-like protein		WWY65185.1	96.60	100	
91	71719_72279	+	hypothetical protein	RuvC-like Holliday junction resolvase	YP_010658999.1	97.31	100	replication and transcription

Table S2 Identification of the bacterial mutation site by DNA sequencing.

ZS-PA-11	Chromosome POS	REF	ALT	TYPE	Samples	Gene	Gene Function	Impact
NODE_23	13889	G	A	SNP	9_6	gtaB	UTP--glucose-1-phosphate uridylyltransferase	HIGH
NODE_32	1146	C	G	SNP	9_1,9_2,9_6,9_7	NODE_32_05998	hypothetical protein	MODERATE
NODE_42	242	A	G	SNP	9_1,9_2,9_6	NODE_42_06008	hypothetical protein	MODERATE
NODE_36	592	T	C	SNP	9_6,9_7	NODE_36_06005	hypothetical protein	MODERATE
NODE_36	718	A	G	SNP	9_6	NODE_36_06005	hypothetical protein	MODERATE
NODE_16	28	T	C	SNP	9_1,9_6	CHR_START-NODE_16_05184	-	MODIFIE
NODE_16	49	G	A	SNP	9_1,9_2,9_6	CHR_START-NODE_16_05184	-	MODIFIE
NODE_32	189	A	G	SNP	9_1,9_7	CHR_START-NODE_32_05998	-	MODIFIE
NODE_34	904	G	A	SNP	9_1,9_2,9_6,9_7	.	-	MODIFIE
NODE_34	1032	C	G	SNP	9_1,9_2,9_6,9_7	.	-	MODIFIE
NODE_34	1444	G	A	SNP	9_2,9_6,9_7	.	-	MODIFIE

NODE_34	1554	T	C	SNP	9_1,9_2,9_6,9_7	.	-	MODIFIE
NODE_37	160	A	G	SNP	9_1,9_2,9_6,9_7	.	-	MODIFIE
NODE_37	199	T	C	SNP	9_1,9_2,9_6,9_7	.	-	MODIFIE
NODE_37	1096	C	T	SNP	9_2,9_7	.	-	MODIFIE
NODE_38	150	G	A	SNP	9_1	CHR_START-NODE_38_06006	-	MODIFIE
NODE_39	132	T	C	SNP	9_1,9_2,9_7	.	-	MODIFIE
NODE_39	696	C	T	SNP	9_1,9_2,9_6,9_7	.	-	MODIFIE
NODE_39	789	T	C	SNP	9_1,9_2,9_7	.	-	MODIFIE
NODE_39	797	A	G	SNP	9_1,9_2,9_7	.	-	MODIFIE
NODE_39	819	C	T	SNP	9_1,9_2,9_6,9_7	.	-	MODIFIE
NODE_41	520	G	C	SNP	9_1,9_2,9_7	NODE_41_06007-CHR_END	-	MODIFIE
NODE_41	521	C	A	SNP	9_1,9_2,9_7	NODE_41_06007-CHR_END	-	MODIFIE
NODE_41	523	T	C	SNP	9_1,9_2,9_7	NODE_41_06007-CHR_END	-	MODIFIE
NODE_41	530	G	A	SNP	9_1,9_2,9_7	NODE_41_06007-CHR_END	-	MODIFIE
NO	534	A	AC	IND	9_1,9	NODE_41	-	MOD

DE_41			G	EL	_2,9_7	_06007-CHR_END		IFIE
NODE_41	537	CGA	C	INDEL	9_1,9_2,9_7	NODE_41_06007-CHR_END	-	MODIFIE
NODE_41	554	T	C	SNP	9_1,9_2,9_7	NODE_41_06007-CHR_END	-	MODIFIE
NODE_41	556	T	C	SNP	9_1,9_2,9_7	NODE_41_06007-CHR_END	-	MODIFIE
NODE_41	578	A	G	SNP	9_1,9_2,9_6,9_7	NODE_41_06007-CHR_END	-	MODIFIE
NODE_42	132	C	T	SNP	9_1,9_2,9_6	CHR_START-NODE_42_06008	-	MODIFIE
NODE_42	156	A	G	SNP	9_1,9_2,9_6	CHR_START-NODE_42_06008	-	MODIFIE
NODE_42	201	G	A	SNP	9_1,9_2,9_6	CHR_START-NODE_42_06008	-	MODIFIE
NODE_42	552	A	G	SNP	9_1,9_2	NODE_42_06008-CHR_END	-	MODIFIE
NODE_42	573	G	A	SNP	9_1,9_2	NODE_42_06008-CHR_END	-	MODIFIE
NODE_45	658	G	A	SNP	9_1,9_2,9_6	.	-	MODIFIE
NODE_49	290	C	G	SNP	9_1,9_6,9_7	.	-	MODIFIE
NODE_51	165	G	C	SNP	9_1,9_2,9_6,9_7	.	-	MODIFIE
NODE_51	167	T	C	SNP	9_1,9_2,9_6,9_7	.	-	MODIFIE

NODE_51	186	G	C	SNP	9_1,9_2,9_6,9_7	.	-	MODIFIE
NODE_51	188	T	G	SNP	9_1,9_2,9_6,9_7	.	-	MODIFIE
NODE_51	198	A	G	SNP	9_1,9_2,9_6,9_7	.	-	MODIFIE
NODE_51	200	C	T	SNP	9_1,9_2,9_6,9_7	.	-	MODIFIE
NODE_51	516	G	GATGTT	INDEL	9_7	.	-	MODIFIE
NODE_52	222	C	G	SNP	9_1,9_2,9_6,9_7	.	-	MODIFIE
NODE_52	408	T	C	SNP	9_1,9_2,9_6,9_7	.	-	MODIFIE
NODE_52	429	G	A	SNP	9_1,9_2,9_6,9_7	.	-	MODIFIE
NODE_43	198	T	C	SNP	9_6	.	-	MODIFIE
NODE_43	301	A	G	SNP	9_6	.	-	MODIFIE
NODE_21	58531	T	C	SNP	9_1,9_2,9_6,9_7	lgrD_2	Linear gramicidin synthase subunit D	LOW
NODE_21	58702	A	G	SNP	9_1,9_2,9_6,9_7	lgrD_2	Linear gramicidin synthase subunit D	LOW
NODE_32	469	C	T	SNP	9_1,9_2,9_6,9_7	NODE_32_05998	hypothetical protein	LOW
NODE_32	933	C	T	SNP	9_1,9_2,9_6,9_7	NODE_32_05998	hypothetical protein	LOW
NO	1017	T	C	SNP	9_1,9	NODE_32	hypothetical	LOW

DE_32					_2,9_6	_05998	protein	
NODE_32	1398	G	A	SNP	9_1,9_2,9_6,9_7	NODE_32_05998	hypothetical protein	LOW
NODE_32	1620	T	C	SNP	9_1,9_2,9_6,9_7	NODE_32_05998	hypothetical protein	LOW
NODE_38	999	C	T	SNP	9_1,9_6,9_7	NODE_38_06006	hypothetical protein	LOW
NODE_41	343	G	A	SNP	9_1,9_2,9_7	NODE_41_06007	hypothetical protein	LOW
NODE_41	346	T	G	SNP	9_1,9_2,9_7	NODE_41_06007	hypothetical protein	LOW
NODE_41	370	G	A	SNP	9_1,9_2,9_7	NODE_41_06007	hypothetical protein	LOW
NODE_42	216	G	A	SNP	9_1,9_2,9_6	NODE_42_06008	hypothetical protein	LOW

Table S3 The defense system of bacteria

Bacteria	Contig	System id	type
ZS-PA-01	ctg00001(1)	ctg00001_PD-T4-7_5	PD-T4-7
	ctg00008(4)	ctg00008_Prithvi_4	Prithvi
		ctg00008_Gabija_1	Gabija
		ctg00008_Gao_Upx_2	Gao_Upx
		ctg00008_Lamassu-Cap4_nuclease_3	Lamassu-Fam
	ctg00021(1)	ctg00021_PD-Lambda-5_6	PD-Lambda-5
ZS-PA-02	ctg00002(1)	ctg00002_PD-T7-3_1	PD-T7-3
	ctg00004(1)	ctg00004_PD-T7-3_2	PD-T7-3
	ctg00008(1)	ctg00008_Gao_Mza_3	Gao_Mza
ZS-PA-03	ctg00004(1)	ctg00004_Gao_Mza_1	Gao_Mza
	ctg00008(2)	ctg00008_Prithvi_2	Prithvi
		ctg00008_Prometheus_3	Prometheus
	ctg00014(1)	ctg00014_Gabija_4	Gabija
ZS-PA-04	ctg00002(1)	ctg00002_DarTG_5	DarTG
	ctg00003(1)	ctg00003_AbiD_4	AbiD
	ctg00006(2)	ctg00006_CAS_Class1-Subtype-I-C_9	Cas
		ctg00006_Gabija_3	Gabija
	ctg00012(2)	ctg00012_pAgo_S2B_2	pAgo
		ctg00012_DS-38_1	DS-38
	ctg00016(1)	ctg00016_CAS_Class1-Subtype-I-F_6	Cas
ZS-PA-05		PA5-chr_RloC_13	RloC
		PA5-chr_Shango_14	Shango
		PA5-chr_AbiE_1	AbiE
		PA5-chr_RM_Type_IIG_2_19	RM
		PA5-chr_Prometheus_12	Prometheus
		PA5-chr_CBASS_III_7	CBASS
		PA5-chr_PD-T4-5_11	PD-T4-5
		PA5-chr_CBASS_II_6	CBASS
		PA5-chr_Gabija_8	Gabija
		PA5-chr_Wadjet_I_16	Wadjet
		PA5-chr_CAS_Class1-Subtype-I-E_21	Cas
		PA5-chr_Hachiman_9	Hachiman
		PA5-chr_RM_Type_I_18	RM
		PA5-chr_RM_Type_IV_20	RM
ZS-PA-07	ctg00001(5)	ctg00001_Retron_III_5	Retron
		ctg00001_RosmerTA_6	RosmerTA
		ctg00001_BREX_I_2	BREX

		ctg00001_PD-T4-8_3	PD-T4-8
		ctg00001_Prometheus_4	Prometheus
	ctg00003(1)	ctg00003_Gabija_7	Gabija
	ctg00008(4)	ctg00008_Zorya_TypeI_18	Zorya
		ctg00008_RM_Type_I_23	RM
		ctg00008_RloC_17	RloC
		ctg00008_DS-1_16	DS-1
	ctg00012(1)	ctg00012_Lamassu-Cap4_nuclease_15	Lamassu-Fam
	ctg00017(4)	ctg00017_CBASS_II_10	CBASS
		ctg00017_Wadjet_I_14	Wadjet
		ctg00017_Gabija_11	Gabija
		ctg00017_RloC_12	RloC
	ctg00022(1)	ctg00022_PD-T7-3_21	PD-T7-3
	ctg00039(1)	ctg00039_Mokosh_Type_I_A_20	Mokosh
ZS-PA-08	ctg00001(1)	ctg00001_RM_Type_II_14	RM
	ctg00002(1)	ctg00002_Nantosuelta_1	Nantosuelta
	ctg00008(2)	ctg00008_DS-32_2	DS-32
		ctg00008_RosmerTA_3	RosmerTA
	ctg00009(1)	ctg00009_Wadjet_II_7	Wadjet
	ctg00013(2)	ctg00013_DRT9_8	DRT
		ctg00013_RM_Type_I_16	RM
	ctg00014(1)	ctg00014_Prometheus_13	Prometheus
	ctg00015(2)	ctg00015_JukAB_9	JukAB
		ctg00015_Wadjet_I_11	Wadjet
	ctg00016(1)	ctg00016_PD-T7-2_4	PD-T7-2
	ctg00021(1)	ctg00021_Gabija_12	Gabija
	ctg00022(1)	ctg00022_RnlAB_5	RnlAB
ZS-PA-09	ctg00007(1)	ctg00007_RM_Type_II_16	RM
	ctg00014(2)	ctg00014_Old_exonuclease_14	Old_exonuclease
		ctg00014_PfiAT_15	PfiAT
	ctg00016(2)	ctg00016_Gabija_11	Gabija
		ctg00016_Wadjet_I_13	Wadjet
	ctg00017(4)	ctg00017_RloC_5	RloC
		ctg00017_Gabija_4	Gabija
		ctg00017_Wadjet_I_7	Wadjet
		ctg00017_CBASS_II_3	CBASS
	ctg00030(1)	ctg00030_RM_Type_IIG_17	RM
	ctg00031(1)	ctg00031_CBASS_III_10	CBASS
ZS-PA-10	ctg00001(1)	ctg00001_Lamassu-Mrr_1	Lamassu-Fam
	ctg00007(1)	ctg00007_Lamassu-Cap4_nuclease_3	Lamassu-Fam
	ctg00008(3)	ctg00008_Prometheus_6	Prometheus

		ctg00008_Septu_7	Septu
		ctg00008_BREX_II_5	BREX
	ctg00012(1)	ctg00012_Gao_Qat_9	Gao_Qat
	ctg00015(1)	ctg00015_gcu24_8	gcu24
	ctg00017(1)	ctg00017_Rst_3HP_2	Rst_3HP
	ctg00024(1)	ctg00024_AbiD_10	AbiD
ZS-PA-11	ctg00001(3)	ctg00001_CBASS_I_5	CBASS
		ctg00001_Lamassu-PDDEXK_7	Lamassu-Fam
		ctg00001_Gao_Mza_6	Gao_Mza
	ctg00004(1)	ctg00004_SspBCDE_1	SspBCDE
	ctg00006(2)	ctg00006_Gabija_2	Gabija
		ctg00006_TIR-III_3	TIR-III
ZS-PA-13	ctg00001(2)	ctg00001_RloC_6	RloC
		ctg00001_Shango_7	Shango
	ctg00002(1)	ctg00002_AbiE_1	AbiE
	ctg00004(2)	ctg00004_CAS_Class1-Subtype-I-E_21	Cas
		ctg00004_Wadjet_I_16	Wadjet
	ctg00008(2)	ctg00008_PD-T4-5_11	PD-T4-5
		ctg00008_CBASS_II_10	CBASS
	ctg00009(3)	ctg00009_RM_Type_IIG_2_20	RM
		ctg00009_Prometheus_5	Prometheus
		ctg00009_CBASS_III_4	CBASS
	ctg00014(1)	ctg00014_Hachiman_12	Hachiman
	ctg00016(2)	ctg00016_RM_Type_I_18	RM
		ctg00016_RM_Type_IV_19	RM
	ctg00027(1)	ctg00027_Gabija_14	Gabija
ZS-PA-14	ctg00001(2)	ctg00001_RloC_6	RloC
		ctg00001_Shango_7	Shango
	ctg00002(1)	ctg00002_AbiE_10	AbiE
	ctg00003(2)	ctg00003_CAS_Class1-Subtype-I-E_21	Cas
		ctg00003_Wadjet_I_9	Wadjet
	ctg00008(2)	ctg00008_CBASS_II_13	CBASS
		ctg00008_PD-T4-5_14	PD-T4-5
	ctg00009(3)	ctg00009_RM_Type_IIG_2_20	RM
		ctg00009_Prometheus_5	Prometheus
		ctg00009_CBASS_III_4	CBASS
	ctg00015(1)	ctg00015_Hachiman_15	Hachiman
	ctg00016(2)	ctg00016_RM_Type_I_18	RM
		ctg00016_RM_Type_IV_19	RM
	ctg00028(1)	ctg00028_Gabija_1	Gabija
ZS-PA-15	ctg00009(1)	ctg00009_Prometheus_5	Prometheus
	ctg00011(1)	ctg00011_CAS_Class1-Subtype-	Cas

		I-F_8	
	ctg00013(1)	ctg00013_PD-Lambda-2_1	PD-Lambda-2
	ctg00018(1)	ctg00018_Borvo_6	Borvo
	ctg00021(2)	ctg00021_PARIS_I_4	Paris
		ctg00021_RM_Type_IV_7	RM
	ctg00027(1)	ctg00027_BREX_I_3	BREX
ZS-PA-16		PA16_PacBio_GAPS6_3	GAPS6
		PA16_PacBio_DS-22_2	DS-22
		PA16_PacBio_ShosTA_4	ShosTA
		PA16_PacBio_RM_Type_II_5	RM
		PA16_PacBio_AbiU_1	AbiU
ZS-PA-17	ctg00002(1)	ctg00002_RM_Type_II_5	RM
	ctg00006(2)	ctg00006_GAPS6_2	GAPS6
		ctg00006_DS-22_1	DS-22
	ctg00011(1)	ctg00011_ShosTA_3	ShosTA
	ctg00020(1)	ctg00020_AbiU_4	AbiU
ZS-PA-19	ctg00002(1)	ctg00002_Gao_Qat_5	Gao_Qat
	ctg00003(1)	ctg00003_dCTPdeaminase_6	dCTPdeaminase
	ctg00010(1)	ctg00010_6A_MBL_1	6A_MBL
	ctg00021(1)	ctg00021_Gabija_3	Gabija
	ctg00036(2)	ctg00036_RM_Type_II_7	RM
		ctg00036_HEC-09_2	HEC-09
	ctg00046(1)	ctg00046_PfiAT_4	PfiAT
ZS-PA-20	ctg00002(1)	ctg00002_RM_Type_IIG_16	RM
	ctg00003(4)	ctg00003_Azaca_4	Azaca
		ctg00003_RM_Type_III_15	RM
		ctg00003_Shedu_6	Shedu
		ctg00003_Shango_5	Shango
	ctg00007(1)	ctg00007_CBASS_III_3	CBASS
	ctg00009(2)	ctg00009_Hachiman_12	Hachiman
		ctg00009_DS-2_11	DS-2
	ctg00026(1)	ctg00026_JukAB_14	JukAB
	ctg00031(1)	ctg00031_Wadjet_I_8	Wadjet
ZS-PA-21	ctg00002(4)	ctg00002_Eleos_1	Eleos
		ctg00002_RM_Type_I_9	RM
		ctg00002_Zorya_TypeI_3	Zorya
		ctg00002_GAPS4_2	GAPS4
	ctg00003(1)	ctg00003_Rst_HelicaseDUF2290_5	Rst_HelicaseDUF2290
	ctg00004(1)	ctg00004_RM_Type_II_12	RM
	ctg00005(1)	ctg00005_VP1851_4	VP1851
	ctg00012(2)	ctg00012_RM_Type_I_11	RM
		ctg00012_CBASS_I_7	CBASS
	ctg00020(1)	ctg00020_CAS_Class1-Subtype-	Cas

		I-F_13	
ZS-PA-22	ctg00002(1)	ctg00002_RM_Type_II_5	RM
	ctg00006(2)	ctg00006_DS-22_1	DS-22
		ctg00006_GAPS6_2	GAPS6
	ctg00011(1)	ctg00011_ShosTA_3	ShosTA
	ctg00020(1)	ctg00020_AbiU_4	AbiU
ZS-PA-23	ctg00002(1)	ctg00002_CBASS_III_3	CBASS
	ctg00003(2)	ctg00003_RM_Type_I_16	RM
		ctg00003_CoCoNut_II_5	CoCoNut
	ctg00012(3)	ctg00012_Pycsar_13	Pycsar
		ctg00012_RM_Type_IIG_18	RM
		ctg00012_RloC_14	RloC
	ctg00016(4)	ctg00016_CBASS_II_10	CBASS
		ctg00016_DISARM_1_11	DISARM
		ctg00016_RM_Type_II_17	RM
		ctg00016_AbiE_7	AbiE
	ctg00019(1)	ctg00019_RosmerTA_4	RosmerTA
	ctg00029(1)	ctg00029_RloC_6	RloC
	ctg00037(1)	ctg00037_Druantia_III_12	Druantia
ZS-PA-24	ctg00001(1)	ctg00001_Lamassu-Mrr_7	Lamassu-Fam
	ctg00007(1)	ctg00007_Lamassu-Cap4_nuclease_8	Lamassu-Fam
	ctg00008(3)	ctg00008_BREX_II_4	BREX
		ctg00008_Septu_6	Septu
		ctg00008_Prometheus_5	Prometheus
	ctg00012(1)	ctg00012_Gao_Qat_1	Gao_Qat
	ctg00015(1)	ctg00015_gcu24_10	gcu24
	ctg00017(1)	ctg00017_Rst_3HP_9	Rst_3HP
	ctg00024(1)	ctg00024_AbiD_2	AbiD
ZS-PA-25	ctg00003(1)	ctg00003_Nantosuelta_2	Nantosuelta
	ctg00004(3)	ctg00004_RM_Type_I_12	RM
		ctg00004_Zorya_TypeI_3	Zorya
		ctg00004_RM_Type_IV_13	RM
	ctg00013(1)	ctg00013_PD-Lambda-5_14	PD-Lambda-5
	ctg00014(2)	ctg00014_Wadjet_I_9	Wadjet
		ctg00014_JukAB_7	JukAB
	ctg00022(1)	ctg00022_RnlAB_1	RnlAB
	ctg00027(2)	ctg00027_Lamassu-Cap4_nuclease_5	Lamassu-Fam
		ctg00027_Hma_4	Hma
	ctg00028(1)	ctg00028_Lamassu-Cap4_nuclease_6	Lamassu-Fam
	ctg00029(1)	ctg00029_RM_Type_III_10	RM
ZS-PA-27	ctg00001(2)	ctg00001_CAS_Class1-Subtype-	Cas

		I-E_10	
		ctg00001_Avs_V_3	Avs
	ctg00002(1)	ctg00002_CBASS_I_2	CBASS
	ctg00008(1)	ctg00008_Prometheus_4	Prometheus
	ctg00016(1)	ctg00016_CAS_Class1-Subtype-I-F_7	Cas
	ctg00021(1)	ctg00021_BREX_I_6	BREX
ZS-PA-28	ctg00002(1)	ctg00002_DarTG_1	DarTG
	ctg00009(1)	ctg00009_Prometheus_4	Prometheus
	ctg00011(1)	ctg00011_PD-Lambda-2_2	PD-Lambda-2
	ctg00012(1)	ctg00012_CAS_Class1-Subtype-I-F_6	Cas
	ctg00017(1)	ctg00017_Borvo_5	Borvo
	ctg00025(1)	ctg00025_DRT_3_3	DRT
ZS-PA-29	ctg00009(1)	ctg00009_Prometheus_1	Prometheus
	ctg00012(1)	ctg00012_CAS_Class1-Subtype-I-F_8	Cas
	ctg00014(1)	ctg00014_PD-Lambda-2_5	PD-Lambda-2
	ctg00019(1)	ctg00019_Borvo_6	Borvo
	ctg00022(2)	ctg00022_PARIS_I_4	Paris
		ctg00022_RM_Type_IV_7	RM
	ctg00027(1)	ctg00027_BREX_I_3	BREX
ZS-PA-30	ctg00001(1)	ctg00001_Lamassu-Mrr_3	Lamassu-Fam
	ctg00007(1)	ctg00007_Lamassu-Cap4_nuclease_9	Lamassu-Fam
	ctg00008(3)	ctg00008_Prometheus_7	Prometheus
		ctg00008_Septu_8	Septu
		ctg00008_BREX_II_6	BREX
	ctg00012(1)	ctg00012_Gao_Qat_4	Gao_Qat
	ctg00015(1)	ctg00015_gcu24_2	gcu24
	ctg00017(1)	ctg00017_Rst_3HP_1	Rst_3HP
	ctg00024(1)	ctg00024_AbiD_10	AbiD
ZS-PA-31	ctg00002(1)	ctg00002_Rst_HelicaseDUF2290_1	Rst_HelicaseDUF2290
	ctg00012(1)	ctg00012_RM_Type_I_20	RM
	ctg00013(1)	ctg00013_RosmerTA_12	RosmerTA
	ctg00019(1)	ctg00019_PD-T7-2_13	PD-T7-2
	ctg00024(3)	ctg00024_RM_Type_IV_1_18	RM
		ctg00024_Zorya_TypeI_2	Zorya
		ctg00024_RM_Type_IIG_2_17	RM
	ctg00026(3)	ctg00026_Azaca_3	Azaca
		ctg00026_Hma_5	Hma
		ctg00026_HEC-08_4	HEC-08
	ctg00030(1)	ctg00030_CAS_Class1-Subtype-	Cas

		I-F_21	
	ctg00031(4)	ctg00031_PD-T7-1_9	PD-T7-1
		ctg00031_Gabija_8	Gabija
		ctg00031_RloC_10	RloC
		ctg00031_RloC_11	RloC
	ctg00039(1)	ctg00039_Wadjet_I_7	Wadjet
ZS-PA-32	ctg00001(1)	ctg00001_PD-T7-2_18	PD-T7-2
	ctg00002(1)	ctg00002_RM_Type_I_23	RM
	ctg00003(1)	ctg00003_Rst_HelicaseDUF2290_1	Rst_HelicaseDUF2290
	ctg00004(2)	ctg00004_RM_Type_I_25	RM
		ctg00004_CoCoNut_II_7	CoCoNut
	ctg00010(4)	ctg00010_AbiE_15	AbiE
		ctg00010_6A_MBL_14	6A_MBL
		ctg00010_Gao_Mza_16	Gao_Mza
		ctg00010_RloC_17	RloC
	ctg00012(1)	ctg00012_CBASS_III_11	CBASS
	ctg00014(1)	ctg00014_Gabija_12	Gabija
	ctg00027(1)	ctg00027_RosmerTA_8	RosmerTA
	ctg00029(1)	ctg00029_CAS_Class1-Subtype-I-F_28	Cas
	ctg00031(3)	ctg00031_RM_Type_IV_1_27	RM
		ctg00031_Zorya_TypeI_2	Zorya
		ctg00031_RM_Type_IIG_2_26	RM
	ctg00036(1)	ctg00036_Wadjet_I_4	Wadjet
	ctg00037(2)	ctg00037_RloC_19	RloC
		ctg00037_RloC_20	RloC
	ctg00047(1)	ctg00047_Gabija_21	Gabija
	ctg00050(1)	ctg00050_PD-T7-1_13	PD-T7-1
	ctg00051(1)	ctg00051_Wadjet_I_6	Wadjet
ZS-PA-33	ctg00002(1)	ctg00002_RM_Type_II_5	RM
	ctg00006(2)	ctg00006_DS-22_1	DS-22
		ctg00006_GAPS6_2	GAPS6
	ctg00011(1)	ctg00011_ShosTA_4	ShosTA
	ctg00020(1)	ctg00020_AbiU_3	AbiU
ZS-PA-34	ctg00001(1)	ctg00001_DRT_2_1	DRT
	ctg00015(1)	ctg00015_Retron_II_2	Retron
ZS-PA-35		Pseudomonas_Druantia_I_2	Druantia
		Pseudomonas_Dsr_I_3	Dsr
		Pseudomonas_RM_Type_IV_5	RM
		Pseudomonas_Avs_II_1	Avs
		Pseudomonas_CAS_Class1-Subtype-I-C_6	Cas
		Pseudomonas_ShosTA_4	ShosTA

Table S4 Bacterial strains and plasmids.

Phage mutant strains and plasmids	Genotype or relevant markers	Reference
E. coli		
SM10 lambda	λ-pir	ATCC® 87450
P. aeruginosa		
Δ*galU*	In-frame deletion of *galU*, *P. aeruginosa*	(11)
Δ*gtaB*	In-frame deletion of *gtaB*, *P. aeruginosa* ZS-PA-11	This study
ZS-PA-01	Initially isolated from Zhongshan Hospital, Shanghai, China.	
ZS-PA-02	Initially isolated from Zhongshan Hospital, Shanghai, China.	(11)
ZS-PA-03	Initially isolated from Zhongshan Hospital, Shanghai, China.	(11)
ZS-PA-04	Initially isolated from Zhongshan Hospital, Shanghai, China.	(11)
ZS-PA-05	Initially isolated from Zhongshan Hospital, Shanghai, China.	(11)
ZS-PA-07	Initially isolated from Zhongshan Hospital, Shanghai, China.	(11)
ZS-PA-08	Initially isolated from Zhongshan Hospital, Shanghai, China.	(11)

ZS-PA-09	Initially isolated from Zhongshan Hospital, Shanghai, China.	(11)
ZS-PA-10	Initially isolated from Zhongshan Hospital, Shanghai, China.	(11)
ZS-PA-11	Initially isolated from Zhongshan Hospital, Shanghai, China.	(11)
ZS-PA-13	Initially isolated from Zhongshan Hospital, Shanghai, China.	(11)
ZS-PA-14	Initially isolated from Zhongshan Hospital, Shanghai, China.	(11)
ZS-PA-15	Initially isolated from Zhongshan Hospital, Shanghai, China.	(11)
ZS-PA-16	Initially isolated from Zhongshan Hospital, Shanghai, China.	(11)
ZS-PA-17	Initially isolated from Zhongshan Hospital, Shanghai, China.	(11)
ZS-PA-19	Initially isolated from Zhongshan Hospital, Shanghai, China.	(11)
ZS-PA-20	Initially isolated from Zhongshan Hospital, Shanghai, China.	(11)
ZS-PA-21	Initially isolated from Zhongshan Hospital, Shanghai, China.	(11)
ZS-PA-22	Initially isolated from Zhongshan Hospital, Shanghai, China.	(11)
ZS-PA-23	Initially isolated from Zhongshan Hospital, Shanghai, China.	(11)
ZS-PA-24	Initially isolated from Zhongshan Hospital, Shanghai, China.	(11)
ZS-PA-25	Initially isolated from Zhongshan Hospital, Shanghai, China.	(11)

ZS-PA-27	Initially isolated from Zhongshan Hospital, Shanghai, China.	(11)
ZS-PA-28	Initially isolated from Zhongshan Hospital, Shanghai, China.	(11)
ZS-PA-29	Initially isolated from Zhongshan Hospital, Shanghai, China.	(11)
ZS-PA-30	Initially isolated from Zhongshan Hospital, Shanghai, China.	(11)
ZS-PA-31	Initially isolated from Zhongshan Hospital, Shanghai, China.	(11)
ZS-PA-32	Initially isolated from Zhongshan Hospital, Shanghai, China.	(11)
ZS-PA-33	Initially isolated from Zhongshan Hospital, Shanghai, China.	(11)
ZS-PA-34	Initially isolated from Zhongshan Hospital, Shanghai, China.	(11)
ZS-PA-35	Initially isolated from Zhongshan Hospital, Shanghai, China.	(11)
plasmids		
pEXG2	Allelic exchange vector with pBR origin, Gm^R, *sacB*	(11)
pΔ*gtaB*	pEXG2, carrying *gtaB* flanking regions, Gm^R	This study
pHB20TG	Arabinose-inducible pBAD promoter, Gm^R	(11)
p*gtaB*	pHB20TG carrying ZS-PA-11 *gtaB*, Gm^R	This study

Table S5 Primers used in the experiments.

**Name**	**Primer (5’-3’)**
*gtaB*_1	TTTAAGCTTGCGACGATTACAGTCATTTCGG
*gtaB*_2	TGTCGAAGGATTCTCGCCTCGCCGTTCCCGATGAAGAACG
*gtaB*_3	CGGGAACGGCGAGGCGAGAATCCTTCGACATGGACGAAGT
*gtaB*_4	TTTTCTAGACGAGCCTGGTCCTGCTGG
*gtaB*_com_1	TTTGAATTCATGATCAAGAAATGTCTTTTCCCG
*gtaB*_com_2	TTTTCTAGATCAGTGAGCCTTGCCGGT
B_1	GCAAGCGCATCGAGTATC
B_2	GGTGCTCAAGACGAACTG
G_1	ATGTAACCCTCGGCGCTA
G_2	CAAGCGTTCGCTGGAAGA
